# Inhibiting 15-PGDH restores redox homeostasis and confers neuroprotection in Parkinson's disease

**DOI:** 10.1016/j.redox.2026.104285

**Published:** 2026-06-30

**Authors:** Young-Kwang Kim, Yun Jae Cha, Se Eun Park, Hee Kyung Kim, Chaesun Kwon, Geonmo Kim, Yoonah R. Oh, Edwin Vázquez-Rosa, Ujjwal Dahiya, Helen Moinova, Yeojung Koh, Farrah Gao, Sunil Jamuna Tripathi, Suwarna Chakraborty, Dong-Gyu Jo, Minseo Woo, Hyejin Park, Seung-Jae Myung, Jiwon Cheon, Yunjong Lee, Xin Qi, Bindu D. Paul, Stephen Fink, Lakshmi Kasturi, James Lutterbaugh, Sanford D. Markowitz, Andrew A. Pieper, Yun Pyo Kang, Min-Kyoo Shin

**Affiliations:** a College of Pharmacy and Research Institute of Pharmaceutical Sciences, Seoul National University, Seoul, Republic of Korea; b Natural Products Research Institute, College of Pharmacy, Seoul National University, Seoul, Republic of Korea; c Innovative Pharmaceutical Sciences Program, College of Transdisciplinary Innovations, Seoul National University, Seoul, Republic of Korea; d Laboratory of Neurodegenerative Diseases, College of Pharmacy, Seoul National University, Seoul, Republic of Korea; e Department of Psychiatry, Case Western Reserve University, Cleveland, OH, USA; f Geriatric Psychiatry, GRECC, Louis Stokes VA Medical Center, Cleveland, OH, USA; g Institute for Transformative Molecular Medicine, School of Medicine, Case Western Reserve University, Cleveland, OH, USA; h Case Comprehensive Cancer Center, Case Western Reserve University, Cleveland, OH, USA; i Department of Medicine, Case Western Reserve University, Cleveland, OH, USA; j Department of Pathology, Case Western Reserve University, Cleveland, OH, USA; k Department of Genetics and Genome Sciences School of Medicine, Case Western Reserve University, Cleveland, OH, USA; l Department of Physiology, Pharmacology and Therapeutics, Johns Hopkins University School of Medicine, Baltimore, MD, USA; m School of Pharmacy, Sungkyunkwan University, Suwon, Republic of Korea; n Graduate School of Medical Science and Engineering, Korea Advanced Institute of Science and Technology, Daejeon, Republic of Korea; o Digestive Diseases Research Center, University of Ulsan College of Medicine, Seoul, Republic of Korea; p Department of Gastroenterology, Asan Medical Center, University of Ulsan College of Medicine, Seoul, Republic of Korea; q Edis Biotech, Songpa-gu, Seoul, Republic of Korea; r Department of Pharmacology, Sungkyunkwan University School of Medicine, Suwon, Republic of Korea; s Department of Physiology and Biophysics, Case Western Reserve University School of Medicine, Cleveland, OH, USA; t Department of Psychiatry and Behavioral Sciences, Johns Hopkins University School of Medicine, Baltimore, MD, USA; u The Solomon H. Snyder Department of Neuroscience, Johns Hopkins University School of Medicine, Baltimore, MD, USA; v Seidman Cancer Center, University Hospitals Cleveland Medical Center, Cleveland, OH, USA; w Brain Health Medicines Center, Harrington Discovery Institute, University Hospitals Cleveland Medical Center, Cleveland, OH, USA; x Department of Neurosciences, Case Western Reserve University, Cleveland, OH, USA

**Keywords:** Parkinson's disease, 15-Hydroxyprostaglandin dehydrogenase, Oxidative stress, Reactive oxygen species, Neuroprotection, SW033291

## Abstract

The prostaglandin- and autocoid-degrading enzyme 15-hydroxyprostaglandin dehydrogenase (15-PGDH) is shown here to be pathologically elevated in Parkinson's disease (PD) patients and mouse models of PD in the substantia nigra, the region of the brain where dopaminergic neurons are lost in PD. Inhibiting 15-PGDH by pharmacologic blockade or partial genetic reduction restores redox homeostasis and mitigates microglial and astrocyte activation, dopaminergic neuron loss, and motor impairment across three mouse models of PD. These models included systemic 1-methyl-4-phenyl-1,2,3,6-tetrahydropyridine (MPTP), intranigral lipopolysaccharide (LPS), and intrastriatal AAV-α-synuclein with intra-ventral tegmental area α-synuclein preformed fibrils (PFFs). The neuroprotective efficacy of 15-PGDH inhibition in PD is shown to be mediated by downregulation of the dopaminergic neuronal cell death mediator lipocalin-2 (*Lcn2*), the pro-inflammatory cytokine interleukin-1β, the reactive oxygen generator *Cybb/No*x2, and oxidative tissue damage. Mechanistically, *in vitro* exposure of BV2 microglia to LPS recapitulates induction of *Lcn2, Cybb*/N OX2 and superoxide, and all three of these effects are reversed by co-treating with prostaglandin E2 (PGE2), the prototypical degradation substrate of 15-PGDH. With a 15-PGDH inhibitor (MF-300) currently in human clinical trials for peripheral indications, these findings have translational relevance for PD.

## 1 Introduction

Parkinson's disease (PD), the second most common neurodegenerative disorder, afflicts millions of individuals, with annual incidence rates ranging from 108 to 212 per 100,000 adults aged 65 and older [1,2]. PD is pathologically defined by progressive degeneration of dopaminergic neurons in the substantia nigra pars compacta (SNpc), resulting in striatal dopamine depletion and motor rigidity, tremors, and bradykinesia [3]. Mainstay therapies such as levodopa transiently alleviate PD symptoms by restoring dopamine levels, but long-term utility is curtailed by diminishing efficacy with disease progression and risk of neuropsychiatric side effects, including hallucinations, delusions, agitation, and impulse control disorders [4]. Unfortunately, no treatments currently halt PD progression. Thus, there is an urgent need to understand and target the root mechanisms of this disease.

The enzyme 15-hydroxyprostaglandin dehydrogenase (15-PGDH) occupies a central regulatory role in bioactive lipid metabolism, catalyzing nicotinamide adenine dinucleotide (NAD^+^)-dependent degradation of prostaglandins and autocoids, with prostaglandin E2 (PGE2) representing its prototypical substrate [[5], [6], [7], [8]]. Our prior work identified SW033291 as a potent small-molecule 15-PGDH inhibitor that enhances tissue regeneration by activating stem cell populations in peripheral organs [6]. Subsequent studies revealed that 15-PGDH inhibition attenuates oxidative stress and inflammation, conferring protection against renal and hepatic injury, albeit by incompletely understood mechanisms [[9], [10], [11]]. More recently, we demonstrated that 15-PGDH suppression counteracts neuroinflammation and blocks generation of myeloid-derived reactive oxygen species (ROS) in the brain, thereby preserving blood-brain barrier (BBB) integrity and preventing neurodegeneration and cognitive impairment in mouse models of traumatic brain injury (TBI) and Alzheimer's disease (AD) [12]. Notably, this neuroprotection occurred without altering amyloid pathology in an amyloid-driven mouse AD model, revealing a paradigm-shifting therapeutic axis for AD [12].

Here, we establish the therapeutic potential of 15-PGDH inhibition in PD, a mechanistically different neurodegenerative disorder. We observed significant 15-PGDH upregulation in the substantia nigra of postmortem human PD brains as well as three different mouse models of PD: systemic 1-methyl-4-phenyl-1,2,3,6-tetrahydropyridine (MPTP) [13], intranigral lipopolysaccharide (LPS) [14], intrastriatal AAV-α-synuclein with intra-ventral tegmental area α-synuclein preformed fibrils (PFFs) [15]. We utilized both pharmacologic inhibition (SW033291 treatment) and genetic partial reduction of 15-PGDH. SW033291 has high specificity for inhibiting 15-PGDH, with IC50 < 1 nM and no interaction with other structurally related short-chain dehydrogenases [6]. Nonetheless, to control for any previously unidentified off-target drug effects, we compared 15-PGDH inhibition with SW033291 versus genetic inhibition of 15-PGDH via knockout of one *Hpgd* allele. Both drug and genetic inhibition of 15-PGDH showed complete concordance in protecting from motor deficits, dopaminergic cell loss, BBB degradation, and induction of multiple molecular markers of inflammation and ROS-induced tissue damage. This identification of SW033291 and genetic reduction of *Hpgd* in protecting the substantia nigra is consistent with our prior findings of complete concordance of SW033291 and genetic deletion of *Hpgd* in AD and TBI models [12].

Transcriptomic profiling via bulk RNA sequencing revealed that 15-PGDH blockade suppresses inflammatory and oxidative stress pathways in the brain, notably reducing levels of mRNA transcripts for the inflammatory cytokine interleukin 1β (*Il1β*), the dopaminergic neuronal cell death driver lipocalin-2 (*Lcn2*). Moreover, *in vivo* 15-PGDH inhibition reduced expression of the reactive oxygen generator *Cybb/*NOX2, reduced generation of chemical superoxide, and preserved BBB integrity. *In vitro*, these effects were recapitulated in activated microglia in which PGE2 treatment attenuated induction of LCN2, blocked induction of *Cybb*/NOX2, and prevented generation of reactive oxygen, with effects on LCN2 dependent signaling via PGE2 receptor 4 (EP4). Thus, we identify 15-PGDH as a master regulator of PD pathophysiology that orchestrates oxidative stress, neuroinflammation, and BBB breakdown as critical events leading to dopaminergic neuronal loss and motor symptoms, and show that 15-PGDH is a therapeutic target whose inhibition mitigates these core pathological drivers of PD.

## 2 Methods

### 2.1 Cell lines

SH-SY5Y, SH-SY5Y α-syn BiFC, and BV2 microglial cells (Elabscience, EP-CL-0493 for NOX2 Western blotting; AcceGen Biotechnology, ABC-TC212S for superoxide experiments) were maintained in Dulbecco's Modified Eagle Medium (DMEM, Welgene, LM 001-05) containing 10% FBS (Gibco, 16000-044) and 1% antibiotic-antimycotic solution (Welgene, LS 203-01) at 37°C under 5% CO_2_. G418 (250 μg/ml) was treated in a medium to keep the selection of α-syn expressing cells.

### 2.2 Animals

Male C57BL/6J mice were purchased from Daehan BioLink Co., Ltd (Korea) and maintained at the Animal Center for Pharmaceutical Research of Seoul National University under temperature, light, and humidity-controlled conditions with free access to food and water. *Hpgd* ^+/-^ males and females were bred and maintained under the same conditions as C57BL/6J mice. All animal work was approved by the Institutional Animal Care and Use Committee (IACUC, SNU-221026-6-5) of Seoul National University.

### 2.3 Human subjects

Studies involving human brain samples were approved by the Institutional Review Board (IRB) from Seoul National University (IRB Number. E2301/001-010).

Additional experimental details (MPTP administration, intranigral LPS administration, stereotaxic injection of α-syn PFF with AAV-human α-syn, stereotaxic injection of α-syn PFF, RT-qPCR, western blotting, 15-PGDH enzyme activity, behavioral analysis, immunohistochemistry, quantification of immunohistochemistry, measurement of MAO-B activity, bulk RNA sequencing, superoxide measurement, electron microscopy, tissue sample preparation for prostaglandin extraction, LC-MS/MS-based prostaglandin analysis, quantification and statistical analysis) are provided in Supplemental Materials.

## 3 Results

### 3.1 15-PGDH is elevated in PD across cellular models, mouse models, and human brain

Previous studies have demonstrated that 15-PGDH inhibition confers protection from liver, colon, bone marrow, and kidney injury through stimulating resident stem and stem-like cells, and protects in TBI and AD through preventing ROS-mediated damage to the BBB [[9], [10], [11], [12],^16^]. To explore a potential role for 15-PGDH in PD, we analyzed 15-PGDH expression in the substantia nigra and striatum in human PD tissues and rodent PD models. The substantia nigra houses the dopaminergic neurons that project axons to the striatum to control movement, and loss of these cells is a principal pathophysiology of PD.

**Fig. 1:**
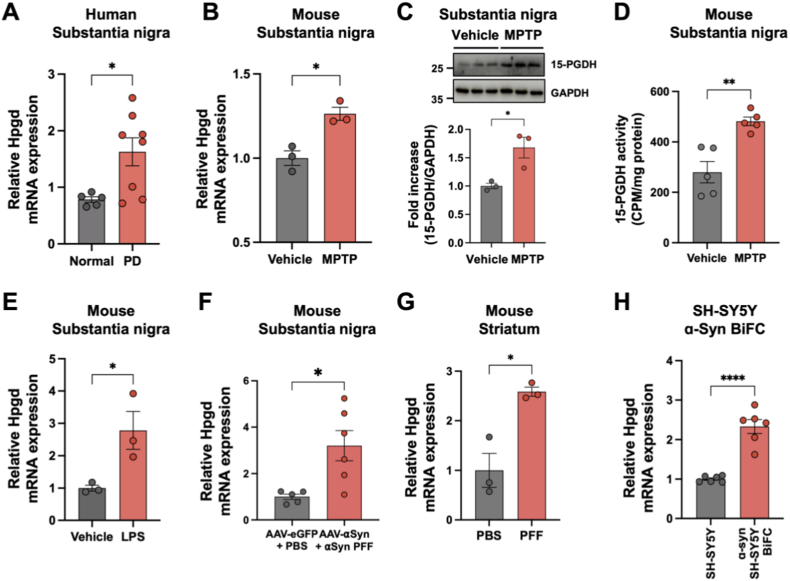
15-PGDH is elevated in Parkinson's disease (PD) in cellular and animal models and human brains (A) *HPGD* mRNA is increased in the substantia nigra of human PD brain, relative to control subjects (n = 5-8 per group, ∗p < 0.05, unpaired *t*-test). (B) *Hpgd* mRNA is increased in the substantia nigra of MPTP-exposed mice, relative to vehicle-treated mice (n = 3 per group, ∗p < 0.05, unpaired *t*-test). (C) 15-PGDH protein expression is increased in the substantia nigra of MPTP-exposed mice, relative to vehicle-treated mice (n = 3 per group, ∗p < 0.05, unpaired *t*-test). (D) 15-PGDH activity is elevated in the substantia nigra of MPTP-exposed mice, relative to vehicle-treated animals (n = 5 per group, ∗∗p < 0.01, unpaired *t*-test). (E) *Hpgd* mRNA expression is elevated in mice expose to intranigral LPS administration, relative to vehicle-treated mice (n = 3 per group, ∗p < 0.05, unpaired *t*-test). (F) *Hpgd* mRNA expression is increased in mice with intrastriatal AAV-ɑ-synuclein combined with intra-ventral tegmental area injection of ɑ-synuclein preformed fibrils (PFF), relative to AAV-eGFP and PBS-exposed mice (n = 5-6 per group, ∗p < 0.05, unpaired *t*-test) (G) *Hpgd* mRNA expression is increased in mice with intrastriatal fibrillar alpha-synuclein administration, relative to PBS-exposed mice (n = 3 per group, ∗p < 0.05, unpaired *t*-test). (H) *HPGD* mRNA is increased in the SH-SY5Y α-syn BiFC cell line, which stably expresses wild-type alpha-synuclein, relative to control SH-SY5Y cells (n = 6 per group, ∗∗∗∗p < 0.0001, unpaired *t*-test).

Postmortem human substantia nigra tissue from PD subjects displayed elevated mRNA for *Hpgd* (the gene encoding 15-PGDH) (Fig. 1A; Table S1). Similarly, exposure of mice to the neurotoxin MPTP, which selectively targets the substantia nigra dopaminergic neurons implicated in PD [[17], [18], [19], [20]], also increased *Hpgd* mRNA levels, 15-PGDH protein levels, and 15-PGDH enzymatic activity in the substantia nigra (Fig. 1B–D). *Hpgd* mRNA was similarly elevated in mice receiving intranigral LPS, intrastriatal AAV-α-synuclein with intra-ventral tegmental area α-synuclein PFFs, and intrastriatal PFF (Fig. 1E–G). Lastly, *Hpgd* mRNA induction was observed in SH-SY5Y cells that were transduced to overexpress wild-type α-synuclein, a cellular model of PD (Fig. 1H). Collectively, our findings establish 15-PGDH elevation as a consistent feature across human PD and preclinical models.

### 3.2 Pharmacologic inhibition and genetic partial reduction of 15-PGDH preserves motor function in mouse PD models

**Fig. 2:**
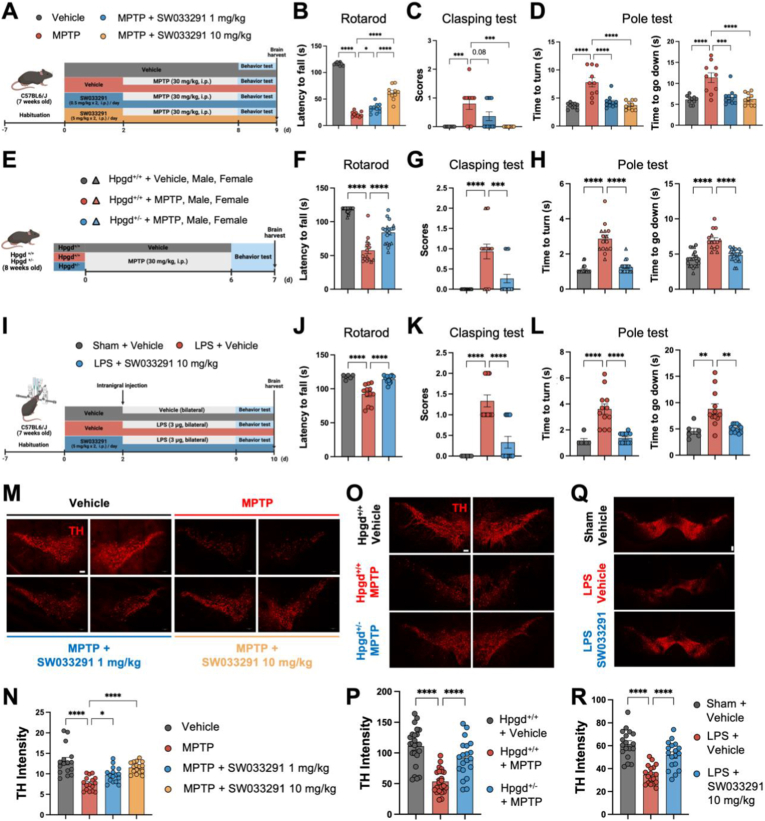
Pharmacological and genetic inhibition of 15-PGDH ameliorates motor deficits and dopaminergic neuronal loss in MPTP- and intranigral LPS-induced PD models (A) Experimental procedure for evaluating neuroprotective efficacy of SW033291 in the MPTP-induced mouse PD model. (B) SW033291 protects MPTP-exposed mice from motor deficits in the rotarod test (n = 10-11 per group, ∗p < 0.05, ∗∗∗∗p < 0.0001, one-way ANOVA and Tukey's post hoc analysis). (C) SW033291 dose-dependently protects MPTP-exposed mice from hindlimb clasping behavior (n = 10-11 per group, ∗∗∗p < 0.001, one-way ANOVA and Tukey's post hoc analysis). (D) SW033291 protects MPTP-exposed mice from increased time to orient downward (time to turn) and total time to descend to the floor (time to go down) in the pole test (n = 10-11 per group, ∗∗∗p < 0.001, ∗∗∗∗p < 0.0001, one-way ANOVA and Tukey's post hoc analysis). (E) Experimental procedure for evaluating neuroprotective efficacy of 15-PGDH haploinsufficiency (*Hpgd*^*+/−*^) in the MPTP-induced mouse PD model. (F) Male and female *Hpgd* heterozygous mice are protected from MPTP-induced motor deficits in the rotarod test (n = 15-22 per group, ∗∗∗∗p < 0.0001, one-way ANOVA and Tukey's post hoc analysis, males and females are shown as circles and triangles, respectively). (G) *Hpgd* heterozygous mice are protected from MPTP-induced hindlimb clasping behavior (n = 15-22 per group, ∗∗∗p < 0.001, ∗∗∗∗p < 0.0001, one-way ANOVA and Tukey's post hoc analysis, males and females are shown as circles and triangles, respectively). (H) *Hpgd* heterozygous mice are protected from MPTP-induced increased time to orient downward (time to turn) and total time to descend to the floor (time to go down) in the pole test (n = 15-22 per group, ∗∗∗∗p < 0.0001, one-way ANOVA and Tukey's post hoc analysis, males and females are shown as circles and triangles, respectively). (I) Experimental procedure for evaluating the neuroprotective efficacy of SW033291 in the intranigral LPS-injected mouse PD model. (J) SW033291 protects mice from intranigral LPS-induced motor deficits in the rotarod test (n = 6-12 per group, ∗∗∗∗p < 0.0001, one-way ANOVA and Tukey's post hoc analysis). (K) SW033291 protects mice from mice from intranigral LPS-induced hindlimb clasping behavior (n = 6-12 per group, ∗∗∗∗p < 0.0001, one-way ANOVA and Tukey's post hoc analysis). (L) SW033291 protects mice from intranigral LPS-induced increased time to orient downward (time to turn) and total time to descend to the floor (time to go down) in the pole test (n = 6-12 per group, ∗∗p < 0.01, ∗∗∗∗p < 0.0001, one-way ANOVA and Tukey's post hoc analysis). (M) Representative TH-stained images of the substantia nigra of mice treated with vehicle or MPTP in the absence or presence of SW033291 (scale bar = 100 μm) (N) Quantification of TH fluorescence signal shows that SW033291 treatment dose-dependently protects MPTP-exposed mice from loss of TH intensity (n = 4 per group, ∗p < 0.05, ∗∗∗∗p < 0.0001, one-way ANOVA and Tukey's post hoc analysis). (O) Representative TH-stained images of the substantia nigra from *Hpgd* heterozygous mice and their wild-type littermates treated with vehicle or MPTP (scale bar = 100 μm) (P) Quantification of TH fluorescence signal shows that *Hpgd* heterozygous mice are protected from MPTP-induced loss of TH intensity (n = 4 per group, ∗∗∗∗p < 0.0001, one-way ANOVA and Tukey's post hoc analysis). (Q) Representative TH-stained images of the substantia nigra from mice treated with vehicle or LPS, in the absence or presence of SW033291 (scale bar = 200 μm) (R) Quantification of TH fluorescence signal shows that SW033291 treatment protects mice from intranigral LPS-induced loss of TH intensity (n = 3 per group, ∗∗∗∗p < 0.0001, one-way ANOVA and Tukey's post hoc analysis).

We next investigated the neuroprotective potential of 15-PGDH inhibition in two PD models, systemic MPTP administration and intranigral LPS injection. In the MPTP study, mice underwent a one-week acclimation period prior to receiving intraperitoneal injections of the 15-PGDH inhibitor SW033291 (0.5 or 5 mg/kg, twice daily) for two days, after which treatment was continued alongside daily MPTP injections (30 mg/kg) for seven more days. SW033291 significantly reduced 15-PGDH activity and increased PGE2 levels in the substantia nigra without affecting body weight (Fig. S1A–C). Behavioral assessments were conducted 2–3 h after the final MPTP injection (Fig. 2A). MPTP administration induced significant motor deficits, evidenced by reduced rotarod latency (Fig. 2B), increased hind-limb clasping (Fig. 2C and Fig. S1D and E), and prolonged pole test completion times (Fig. 2D). Notably, SW033291 treatment dose-dependently prevented all these impairments, preserving motor performance to near non-MPTP exposed levels (Fig. 2B–D and Fig. S1E).

We next examined whether genetic partial reduction of 15-PGDH activity could also provide neuroprotection against MPTP-induced behavioral deficits. Eight-week-old male and female *Hpgd*^*+/+*^ (wild type: WT) and *Hpgd*^*+/−*^ (haploinsufficient) mice received daily MPTP injections (30 mg/kg) for seven days, followed by behavioral assessments 2-3 h after the final injection (Fig. 2E). MPTP did not affect body weight in either genotype (Fig. S1F). After MPTP exposure, heterozygous *Hpgd* ^*±/-*^ mice exhibited superior motor performance compared to WT *Hpgd*^*+/+*^ littermates, demonstrating longer rotarod retention (Fig. 2F), reduced hindlimb clasping (Fig. 2G and Fig. S1G), and faster pole test completion (Fig. 2H).

To rule out confounding effects of 15-PGDH inhibition on monoamine oxidase-B (MAO-B), the enzyme that converts MPTP to its neurotoxic metabolite MPP^+^, we measured MAO-B activity and observed that pharmacologic inhibition and genetic partial reduction of 15-PGDH did not affect MAO-B activity in the substantia nigra or striatum (Fig. S1H–K).

We next assessed efficacy in a second PD model: intranigral LPS administration [14,21,22]. Mice pretreated with SW033291 (5 mg/kg, twice daily) or vehicle received bilateral substantia nigra LPS injections, with continued SW033291 treatment (Fig. 2I). LPS administration did not affect body weight (Fig. S1L). LPS impaired motor function one week later, indicated by reduced latency to fall on the accelerating rotarod (Fig. 2J), increased hind-limb clasping (Fig. 2K and Fig. S1M), and prolonged pole test duration (Fig. 2L). Notably, SW033291 prevented these deficits (Fig. 2J–L).

Taken together, our data demonstrates that both pharmacological inhibition (SW033291) and genetic partial reduction (*Hpgd*^*+/−*^*)* of 15-PGDH attenuates PD-associated motor dysfunction in two different preclinical models of PD, supporting 15-PGDH as a therapeutic target for this disease. The efficacy noted in heterozygous 15-PGDH deficient mice underscores the therapeutic potential of this approach, in that only partial 15-PGDH inhibition was necessary for neuroprotective effect.

### 3.3 Pharmacologic inhibition and genetic partial reduction of 15-PGDH prevents dopaminergic neuronal loss in mouse PD models

We next evaluated whether these same interventions also conferred neuroprotection to dopaminergic neurons in these models. Immunohistochemical analysis revealed a significant reduction in tyrosine hydroxylase (TH), a marker of dopaminergic neurons, in the substantia nigra of MPTP-treated mice compared to vehicle-treated controls (Fig. 2M and N). Strikingly, administration of the 15-PGDH inhibitor SW033291 enhanced dopaminergic neuron survival in a dose-dependent manner (Fig. 2M and N). MPTP-injected animals also exhibited pronounced neuronal loss in the striatum, which was dose-dependently attenuated by 15-PGDH inhibition (Fig. S2A and B). Western blot analysis confirmed these findings, showing that 15-PGDH inhibition significantly preserved TH expression in both the substantia nigra and striatum of MPTP-exposed mice (Fig. S2C and D). *Hpgd* ^*+/-*^ mice were also protected from MPTP-induced dopaminergic neuronal loss (Fig. 2O and P, Fig. S2E and F). Western blot analysis confirmed TH levels, which declined in MPTP-exposed *Hpgd*^*+/+*^ mice and remained at baseline in *Hpgd* ^*+/-*^ mice (Fig. S2G and H). Lastly, 15-PGDH inhibition also maintained TH levels in the intranigral LPS model (Fig. 2Q and R).

### 3.4 Bulk RNA sequencing reveals suppression of reactive oxygen species and neuroinflammatory-related pathways by 15-PGDH inhibition

To investigate the neuroprotective mechanism of 15-PGDH inhibition in PD, we performed bulk RNA sequencing of the substantia nigra from SW033291-treated versus vehicle-treated mice in both the MPTP and intranigral LPS-induced PD models. Joint analysis of the SW033291 effect across both models revealed that 15-PGDH inhibition upregulated 126 genes (p < 0.05 and Log2(FC) > 0.5) and downregulated 367 genes (p < 0.05 and Log2(FC) < -0.5) (Fig. S3A; Table S2). Gene Ontology (GO) analysis of biological processes demonstrated significant SW033291 upregulated gene associated processes linked to cell projection organization and neurogenesis (Fig. S3B; Table S3, which highlights the top biological processes with q values of 1.6e-4 to 2.6e-4). GO analysis also identified multiple SW033291 downregulated gene-associated biological processes, at even greater levels of statistical significance, linked to ROS metabolism, inflammation, and immune response regulation (Fig. S3C; Table S4, which highlights selected representative processes with q values of 1.65e-23 to 3.56e-14).

Inspection of GO biological processes associated with SW033291 downregulated genes (Fig. S3C; Table S4) and of individual genes downregulated by SW033291 (Fig. S3A and D; Table S2) identified SW033291 targeting of several highly pathogenic known disease mediators. First, SW033291 treatment decreased levels of both *Cybb* and *Cyba*, which respectively encode gp91^phox^ (commonly called NOX2) and gp22^phox^, cooperating subunits of the ROS generating NADPH oxidase complex [23]. SW033291 treatment also decreased expression of *Lcn2*, an inflammation-associated protein that mediates dopaminergic neuronal death and glial activation [[24], [25], [26]]. Furthermore, 15-PGDH inhibition markedly reduced the levels of the pro-inflammatory cytokine gene *Il1β*. While modulation of each of these gene targets was individually statistically significant (Fig. S3A), they did not reach a threshold for FDR, multiple testing corrected, significance (Table S2). Accordingly, to further investigate these candidate SW033291 effectors, we generated samples from new and independent validation sets of animals, as shown below.

### 3.5 15-PGDH inhibition mitigates oxidative stress in PD models by suppressing NOX2-dependent pathways

**Fig. 3:**
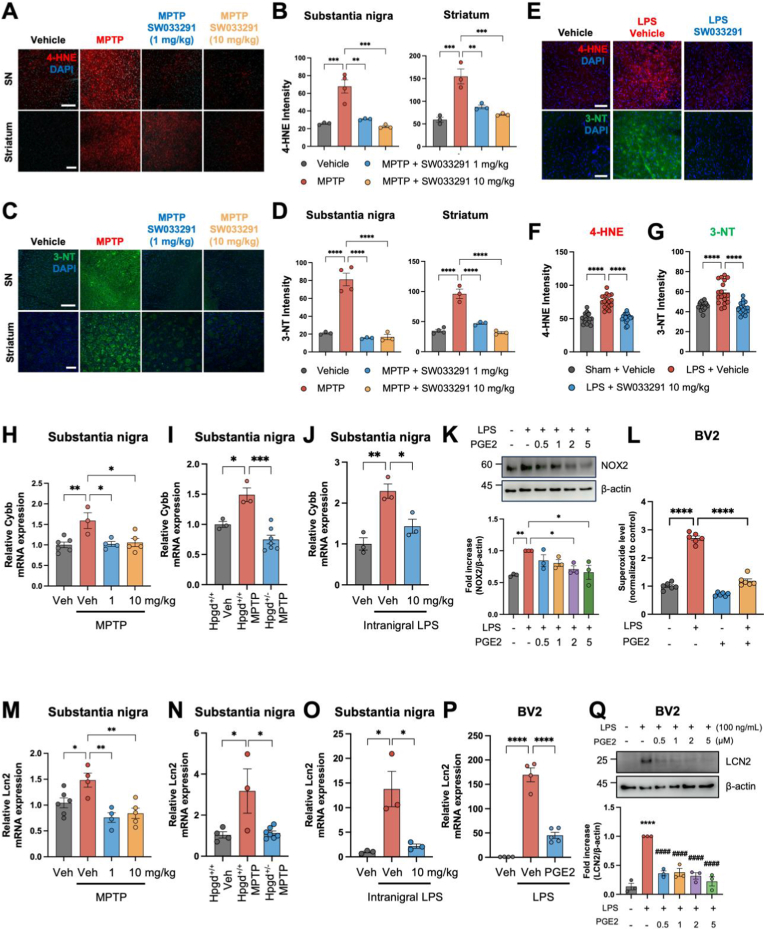
15-PGDH inhibition alleviates oxidative damage and LCN2 expression in PD models (A) Representative 4-HNE stained images of the substantia nigra and striatum of mice treated with vehicle or MPTP in the absence or presence of SW033291 (scale bar = 100 μm) (B) Quantification of 4-HNE fluorescence signal shows that SW033291 treatment reduces mean fluorescence intensity in the substantia nigra and striatum of mice exposed to MPTP (n = 4 per group, ∗∗p < 0.01, ∗∗∗p < 0.001, ∗∗∗∗p < 0.0001, one-way ANOVA and Tukey's post hoc analysis). (C) Representative 3-nitrotyrosine stained images of the substantia nigra and striatum of mice treated with vehicle or MPTP in the absence or presence of SW033291 (scale bar = 100 μm). (D) Quantification of 3-nitrotyrosine fluorescence signal shows that SW033291 treatment reduces mean fluorescence intensity in the substantia nigra and striatum of mice exposed to MPTP (n = 4 per group, ∗∗p < 0.01, ∗∗∗p < 0.001, ∗∗∗∗p < 0.0001, one-way ANOVA and Tukey's post hoc analysis). (E) Representative 4-HNE and 3-nitrotyrosine stained images of the substantia nigra from mice treated with either vehicle or SW033291 in the intranigral LPS model (scale bar = 100 μm) (F) Quantification of 4-HNE fluorescence signal shows that SW033291 treatment reduces fluorescence intensity in the intranigral LPS model (n = 3 per group, ∗∗∗∗p < 0.0001, one-way ANOVA and Tukey's post hoc analysis). (G) Quantification of 3-nitrotyrosine fluorescence signal reveals that SW033291 treatment reduces fluorescence intensity in the intranigral LPS model (n = 3 per group, ∗∗∗∗p < 0.0001, one-way ANOVA and Tukey's post hoc analysis). (H) RT-qPCR analysis shows that SW033291 treatment reduces *Cybb* mRNA expression in the substantia nigra of mice exposed to MPTP (n = 3-6 per group, ∗p < 0.05, ∗∗p < 0.01, one-way ANOVA and Tukey's post hoc analysis). (I) RT-qPCR analysis shows that MPTP-exposed *Hpgd* heterozygous mice have lower *Cybb* levels in the substantia nigra than MPTP-exposed wild-type littermates (n = 3-7 per group, ∗p < 0.05, ∗∗∗p < 0.001, one-way ANOVA and Tukey's post hoc analysis). (J) RT-qPCR analysis shows that SW033291 treatment reduces *Cybb* mRNA expression in the substantia nigra of LPS-treated brains (n = 3 per group, ∗p < 0.05, ∗∗p < 0.01, one-way ANOVA and Tukey's post hoc analysis). (K) NOX2 Western blot and its quantification show that 1 and 5 μM of PGE2 pretreatment significantly decreases NOX2 expression in LPS-treated BV2 microglial cells (n = 3 per group, ∗p < 0.05, ∗∗p < 0.01, one-way ANOVA and Tukey's post hoc analysis). (L) PGE2 (5 μM) treatment significantly reduces superoxide levels in LPS-treated BV2 microglial cells (n = 6 per group, ∗∗∗∗p < 0.0001, one-way ANOVA and Tukey's post hoc analysis). (M) RT-qPCR analysis shows that *Lcn2* is upregulated in MPTP-treated substantia nigra, and that this increase is attenuated by treatment with 1 and 10 mg/kg of SW033291 (n = 4-6 per group, ∗p < 0.05, ∗∗p < 0.01, one-way ANOVA and Tukey's post hoc analysis). (N) RT-qPCR analysis shows that MPTP-exposed *Hpgd* heterozygous mice have lower *Lcn2* levels than MPTP-exposed wild-type littermates (n = 3-7 per group, ∗p < 0.05, one-way ANOVA and Tukey's post hoc analysis). (O) RT-qPCR analysis shows that SW033291 reduces *Lcn2* mRNA expression in intranigral LPS-exposed brain (n = 3 per group, ∗p < 0.05, one-way ANOVA and Tukey's post hoc analysis). (P) RT-qPCR analysis shows that PGE2 treatment significantly reduces *Lcn2* mRNA expression in LPS-exposed BV2 microglia cells (n = 4-5 per group, ∗∗p < 0.01, ∗∗∗∗p < 0.0001, one-way ANOVA and Tukey's post hoc analysis). (Q) Western blot and its quantification show that PGE2 treatment decreases LCN2 levels in LPS-exposed BV2 microglia cells (n = 3 per group, ∗∗∗∗p < 0.0001, one-way ANOVA and Tukey's post hoc analysis).

Oxidative stress is a well-established feature of PD [27,28], with postmortem analyses of PD patient brains demonstrating elevated oxidative stress markers, including 4-hydroxyl-2-nonenal (4-HNE; a lipid peroxidation product) and 3-nitrotyrosine (3-NT; a tyrosine nitration product), particularly in the substantia nigra [29,30]. Elevated 4-HNE levels have also been detected in the cerebrospinal fluid and plasma of PD patients, further implicating oxidative damage in disease progression [31]. Intriguingly, our bulk RNA-seq analysis showed that pharmacologic inhibition of 15-PGDH downregulated ROS-related genes, suggesting that 15-PGDH inhibition might block PD-associated oxidative stress. Accordingly, we found that pharmacologically inhibiting (SW033291) or partially genetically reducing 15-PGDH markedly reduced 4-HNE and 3-NT accumulation in the substantia nigra and striatum of MPTP mice (Fig. 3A–D and Fig. S4A–D). SW033291 treatment also suppressed oxidative stress in the intranigral LPS model, as evidenced by similarly normalizing 4-HNE and 3-NT levels (Fig. 3E–G).

Mechanistically, our bulk RNA-seq data suggested SW033291 suppressed expression of *Cybb*, which encodes the NOX2 catalytic subunit of the NADPH oxidase complex (Fig. S3A and D), a critical superoxide-generating enzyme. Given that NOX2 is upregulated in microglia of the substantia nigra of both human PD subjects and experimental models, including the MPTP model [32], we further interrogated this ROS generating target. In murine models, we found that both MPTP administration and intranigral LPS injection significantly increased *Cybb* mRNA levels in the substantia nigra, which was prevented by pharmacologic inhibition and genetic partial reduction of 15-PGDH (Fig. 3H–J).

To further delineate the basis of these 15-PGDH pathway driven effects, we evaluated regulation of NOX2 expression and superoxide production in BV2 murine microglial cells. PGE2, the prototypical 15-PGDH degradation substrate, dose-dependently inhibited LPS-induced NOX2 upregulation (Fig. 3K). Furthermore, PGE2 also completely inhibited LPS-induced generation of superoxide (Fig. 3K), that was fully derived from NOX2, as shown by its blockade by the NOX2 inhibitor GSK2795039 (Fig. S4E).

These results demonstrate that 15-PGDH inhibition reduces oxidative stress in PD models by suppressing NOX2 expression.

### 3.6 15-PGDH inhibition suppresses microglia and astrocyte activation in PD models

Given that bulk RNA sequencing analysis revealed the suppression of neuroinflammatory responses by 15-PGDH inhibition, we next investigated its effects on microglial and astrocytic activation in PD models. Immunofluorescence analysis demonstrated a marked increase in ionized calcium-binding adapter molecule 1 (Iba1) intensity, a microglial marker, in the substantia nigra and striatum of MPTP-exposed mice, which was attenuated by SW033291 (Fig. S5A–D). To assess astrocyte reactivity, we analyzed glial fibrillary acidic protein (GFAP) expression, a canonical marker of reactive astrocytes [33], and observed that 15-PGDH inhibition also dose-dependently reduced GFAP-positive astrocytes in these regions in MPTP-exposed mice (Fig. S5E–H). Critically, 15-PGDH inhibition also suppressed these markers of microglial and astrocytic activation in the intranigral LPS model (Fig. S5I–L).

We further evaluated genetic modulation of 15-PGDH by comparing *Hpgd*^*+/+*^ and *Hpgd*^+/-^ mice. MPTP exposure robustly elevated Iba1 and GFAP levels in *Hpgd*^*+/+*^ mice compared to vehicle-treated controls, but not in *Hpgd* ^+/-^ mice (Fig. S5M–T). Taken together, our findings demonstrate that 15-PGDH inhibition mitigates neuroinflammatory responses in PD, attenuating both microglial and astrocytic activation.

### 3.7 15-PGDH inhibition attenuates lipocalin-2 expression via PGE2-EP4 signaling

Activated microglia and astrocytes express and secrete LCN2, a mediator of selective dopaminergic neuronal death and glial reactivation [[24], [25], [26]]. To further explore the downregulation of *Lcn2* expression observed in bulk RNA-seq analysis following SW033291 treatment, we evaluated LCN2 levels in an independent repeat of the MPTP PD model. Consistent with prior findings [25], MPTP administration significantly elevated *Lcn2* mRNA and protein levels in the substantia nigra and striatum. By contrast, pharmacologic inhibition (via SW033291) or genetic haploinsufficiency of 15-PGDH suppressed MPTP-induced increase in *Lcn2* expression (Fig. 3M and N and Fig. S6A-D). Notably, SW033291 treatment also attenuated *Lcn2* upregulation in the intranigral LPS model (Fig. 3O).

To elucidate the mechanistic basis of 15-PGDH inhibition in modulating *Lcn2* expression, we treated BV2 microglia cells with PGE2 prior to LPS exposure, a known inducer of *Lcn2* [34]. PGE2 prevented LPS-induced increase in both *Lcn2* mRNA and protein levels (Fig. 3P and Q). Notably, PGE2 suppressed LCN2 at all tested concentrations (0.5, 1, 2, and 5 μM) (Fig. 3Q).

PGE2 signals through four receptor types: EP1, EP2, EP3, and EP4 [35]. To determine which receptor mediates the protective effect of PGE2, we employed receptor-specific antagonists. While EP1, EP2, and EP3 antagonists failed to block PGE2's suppression of LCN2, the EP4-specific antagonist L-161982 dose-dependently inhibited the protective effect of PGE2 on LCN*2* expression in BV2 cells exposed to LPS (Fig. S6E–H). These results demonstrate that PGE2 attenuation of LPS-driven LCN2 expression in microglia is predominantly via the PGE2-EP4 signaling pathway.

### 3.8 15-PGDH inhibition prevents BBB damage in PD models

BBB dysfunction is a hallmark of PD, driven in part by oxidative stress and neuroinflammation in both human and mouse models [36]. Neuroimaging studies further corroborate BBB impairment in the substantia nigra of human PD patients [37,38]. Building on prior evidence that 15-PGDH inhibition mitigates BBB disruption in TBI and AD [12], we investigated whether 15-PGDH blockade could similarly protect BBB integrity in the MPTP mouse model of PD.

Electron microscopy revealed that pharmacological inhibition of 15-PGDH with SW033291 significantly reduced MPTP-induced structural damage to capillary endothelia in the substantia nigra and striatum (Fig. S7A). To confirm functional BBB compromise, we assessed parenchymal infiltration of endogenous immunoglobulin G (IgG), a marker of barrier leakage. MPTP administration markedly increased IgG deposition in brain tissue, an effect fully abrogated by SW033291 treatment (Fig. S7B).

We next evaluated whether genetic inhibition of 15-PGDH could confer similar protection. Electron microscopy again showed severe BBB ultrastructural damage in MPTP-exposed *Hpgd*^*+/+*^ mice, whereas *Hpgd* ^*+/-*^ mice retained intact barrier morphology in both brain regions (Fig. S7C). Consistent with these findings, MPTP triggered robust IgG extravasation in the substantia nigra and striatum of *Hpgd*^*+/+*^ mice, whereas this effect was markedly reduced in *Hpgd* ^*+/-*^ mice (Fig. S7D).

### 3.9 15-PGDH inhibition ameliorates motor deficits, dopaminergic neuronal loss, and oxidative stress without affecting ɑ-synuclein pathology in ɑ-synuclein mouse models

**Fig. 4:**
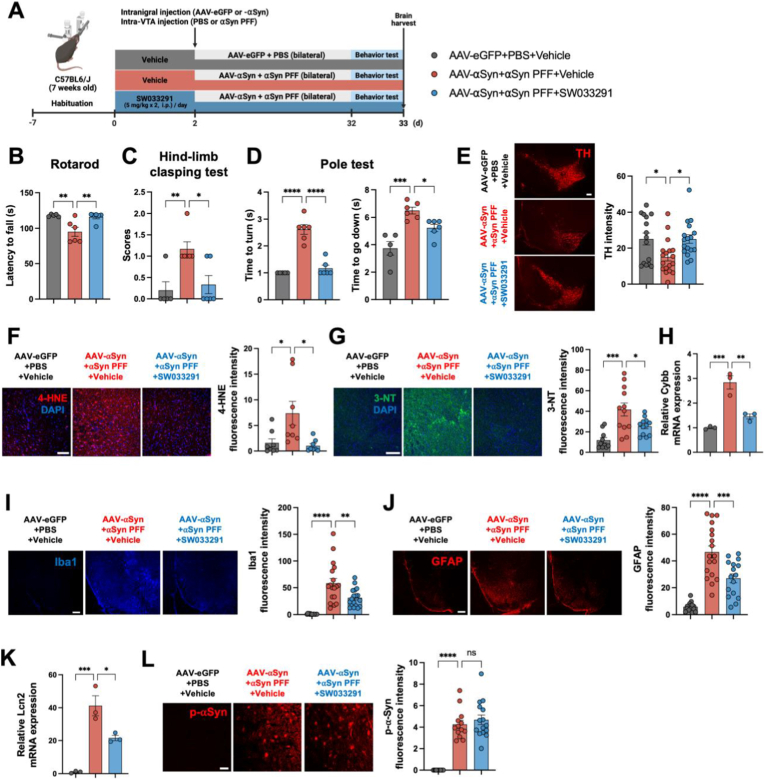
15-PGDH inhibition ameliorates motor deficits, dopaminergic neuronal loss, and oxidative stress in the ɑ-synuclein mouse PD model (A) Experimental procedure for evaluating the efficacy of SW033291 in the α-synuclein mouse model. (B) SW033291 protects mice from AAV-ɑ-synuclein/PFF-induced motor deficits in the rotarod test (n = 5-6 per group, ∗∗p < 0.01, one-way ANOVA and Tukey's post hoc analysis). (C) SW033291 protects mice from AAV-ɑ-synuclein/PFF-induced from hindlimb clasping behavior (n = 5-6 per group, ∗p < 0.05, ∗∗p < 0.01, one-way ANOVA and Tukey's post hoc analysis). (D) SW033291 protects mice from AAV-ɑ-synuclein/PFF-induced increased time to orient downward (time to turn) and total time to descend to the floor (time to go down) in the pole test (n = 5-6 per group, ∗∗∗p < 0.001, ∗∗∗∗p < 0.0001, one-way ANOVA and Tukey's post hoc analysis). (E) Representative images and quantification of TH immunoreactivity in the substantia nigra of mice treated with vehicle or SW033291. SW033291 administration protected mice from AAV-ɑ-synuclein/PFF-induced loss of TH intensity (scale bar = 800 μm, n = 5-6 per group, ∗p < 0.05, one-way ANOVA and Tukey's post hoc analysis). (F) Representative images and quantification of 4-HNE immunoreactivity in the substantia nigra of mice treated with vehicle or SW033291. SW033291 administration protected mice from AAV-ɑ-synuclein/PFF-induced increase in 4-HNE (scale bar = 100 μm, n = 5-6 per group, ∗p < 0.05, one-way ANOVA and Tukey's post hoc analysis). (G) Representative images and quantification of 3-NT immunoreactivity in the substantia nigra of mice treated with vehicle or SW033291. SW033291 administration protected mice from AAV-ɑ-synuclein/PFF-induced increase in 3-NT (scale bar = 100 μm, n = 5-6 per group, ∗p < 0.05, ∗∗∗p < 0.001, one-way ANOVA and Tukey's post hoc analysis). (H) RT-qPCR analysis shows that SW033291 treatment protected mice from administration protected mice from AAV-ɑ-synuclein/PFF-induced increase in *Cybb* mRNA expression (n = 3 per group, ∗∗p < 0.01, ∗∗∗p < 0.001, one-way ANOVA and Tukey's post hoc analysis). (I) Representative images and quantification of Iba1 immunoreactivity in the substantia nigra of mice treated with vehicle or SW033291. SW033291 administration protected mice from AAV-ɑ-synuclein/PFF-induced increase in Iba1 (scale bar = 200 μm, n = 5-6 per group, ∗∗p < 0.01, ∗∗∗p < 0.001, one-way ANOVA and Tukey's post hoc analysis). (J) Representative images and quantification of GFAP immunoreactivity in the substantia nigra of mice treated with vehicle or SW033291. SW033291 administration protected mice from AAV-ɑ-synuclein/PFF-induced increase in GFAP (scale bar = 300 μm, n = 5-6 per group, ∗∗∗p < 0.001, ∗∗∗∗p < 0.0001, one-way ANOVA and Tukey's post hoc analysis). (K) RT-qPCR analysis shows that SW033291 administration protected mice from AAV-ɑ-synuclein/PFF-induced increase in *Lcn2* mRNA expression (n = 3 per group, ∗p < 0.05, ∗∗∗p < 0.001, one-way ANOVA and Tukey's post hoc analysis). (L) Representative images and quantitative analysis of p-α-synuclein immunoreactivity in the substantia nigra of mice treated with vehicle or SW033291. SW033291 treatment had no effect on p-α-synuclein accumulation in AAV-ɑ-synuclein/PFF mice (scale bar = 20 μm, n = 5-6 per group, ∗∗∗∗p < 0.0001, one-way ANOVA and Tukey's post hoc analysis).

As α-synuclein–driven models most closely recapitulate key features of human PD, we further tested the efficacy of 15-PGDH inhibition in this mouse model. Seven-week-old C57BL/6J mice were acclimated for one week and then pretreated with either vehicle or the 15-PGDH inhibitor SW033291 (5 mg/kg, intraperitoneally, twice daily) for two days prior to surgery. Mice subsequently received intranigral injections of either AAV-GFP (comparator) or AAV–α-synuclein (disease model), followed by intra-VTA injection of PBS (comparator) or α-synuclein preformed fibrils (PFFs) (disease model). Behavioral assessments were performed at designated time points, and brains were collected for biochemical and histological analysis at 33 days post-injection (Fig. 4A). Consistent with our previous reports [39,40], the combination of AAV-α-synuclein and PFF administration induced pronounced motor dysfunction, reflected by shortened rotarod latency (Fig. 4B), elevated hind-limb clasping scores (Fig. 4C), and prolonged pole test times (Fig. 4D). SW033291 treatment prevented these impairments and maintained motor performance comparable to AAV-eGFP/PBS controls, with no change in body weight (Fig. 4B–D and Fig. S8A).

We next examined neuroprotective effects on dopaminergic neurons and reduced oxidative stress in this model. TH, 4-HNE, and 3-NT immunostaining demonstrated that combined administration of AAV-α-synuclein and PFF resulted in decreased TH intensity and elevated oxidative stress markers in the substantia nigra, with SW033291 treatment markedly attenuating these alternations (Fig. 4E–G) along with markedly reducing *Cybb* mRNA levels (Fig. 4H).

To further determine the effect of SW033291 on neuroinflammatory responses and α-synuclein pathology in this model, we performed immunohistochemical analyses for microglial and astrocytic activation, as well as phosphorylated α-synuclein accumulation. Iba1 immunoreactivity, a marker of microglial activation, was markedly increased in the AAV-α-syn + PFF-treated mice compared with control animals. Treatment with SW033291 significantly reduced Iba1 intensity, indicating attenuation of microglial activation (Fig. 4I). Similarly, GFAP staining revealed robust astrocyte activation that was prevented by SW033291 (Fig. 4J). In addition, *Lcn2* expression was significantly elevated in this model and markedly reduced by SW033291 (Fig. 4K).

Notably, SW033291 treatment did not significantly reduce phosphorylated α-synuclein accumulation (Fig. 4L), indicating that 15-PGDH inhibition primarily alleviates oxidative stress and neuroinflammation independent of α-synuclein pathology, and that this is sufficient to eliminate the motor impairment associated with PD. This finding draws a notable parallel with our recent report demonstrating the ability of 15-PGDH inhibition to prevent pathological features and cognitive impairment in an amyloid-driven mouse model of AD without affecting amyloid pathology [12].

## 4 Conclusions and clinical implications

Following the seminal discovery of 15-PGDH's role in promoting tissue repair in hepatic, bone marrow, and colonic injury models [6], its inhibition has emerged as a protective strategy across diverse pathologies, including pulmonary fibrosis [41], renal injury [9,10,16], cardiac fibrosis [42], type 2 diabetes [43,44], TBI [12], and AD [12]. Although the therapeutic mechanisms vary across these diseases, each reflects 15-PGDH's fundamental role in autocoid homeostasis. Here, we establish for the first time that both pharmacologic inhibition and genetic partial reduction of 15-PGDH confer neuroprotection in multiple preclinical PD models. Our results indicate that 15-PGDH inhibition orchestrates a coordinated program of tissue regeneration and repair, though the underlying mechanisms are highly tissue specific. In the bone marrow, for example, protection is driven primarily by stem cell upregulation, whereas in the brain it centers on suppression of oxidative stress and inflammatory cell effectors.

Elevated 15-PGDH levels were observed in both *in vitro* and *in vivo* PD models, as well as in postmortem human PD brain tissue. Both pharmacologic inhibition and genetic partial reduction of 15-PGDH mitigated motor deficits, oxidative damage, neuroinflammation, and dopaminergic neuron loss across MPTP, intranigral LPS, and α-synuclein PD mouse models. Notably, 15-PGDH-mediated protection in the α-synuclein PD mouse model occurred without any change in phosphorylated α-synuclein accumulation, demonstrating that therapeutic benefit can be achieved independently of this aspect of synuclein pathology. This parallels our recent finding that 15-PGDH-mediated neuroprotection in an amyloid-based AD mouse model was similarly independent of alteration in amyloid pathology [12], contributing to a growing body of evidence that potent therapeutic effect can be achieved by targeting the brain's damage and inflammatory response to the classically considered primary drivers of disease [[45], [46], [47]]. Mechanistically, bulk RNA-seq and confirmatory studies revealed that 15-PGDH inhibition downregulates genes associated with oxidative stress and neuroinflammation, including *Cybb, Lcn2,* and *Il1β*. Consistent with these findings, 15-PGDH inhibition suppressed microglial and astrocytic activation and protected against oxidative damage in PD models.

Prior studies have established that 15-PGDH inhibition elevates PGE2 levels [6], which signals via four distinct receptors: EP1, EP2, EP3, and EP4. Here, we found that PGE2, acting through the EP4 receptor, suppresses expression of *Lcn2*, a known mediator of dopaminergic cell death, in LPS-stimulated microglia. This is consistent with reports demonstrating that EP4 receptor activation protects substantia nigra dopaminergic neurons in the MPTP model and that microglia EP4 deletion worsens MPTP-induced neuropathology [48], together supporting a neuroprotective role for the PGE2-EP4-LCN2 inhibitory axis. This regulation may involve EP4 receptor-associated protein (EPRAP), which stabilizes p105 to inhibit NF-kB in bone marrow-derived macrophages [49], a pathway that will be a future area of investigation.

Future exploration of the downstream signaling pathways will likely yield important insights into the physiological and pathological roles of this newly-established PGE2-EP4-LCN2 axis. EP4 signaling can also activate MAPK cascades that regulate cell proliferation, stress responses, and cytokine production, potentially acting in concert with or independently of NF-κB to modulate *Lcn2* expression and broader inflammatory responses. Additionally, NRF2, a master regulator of antioxidant defense and redox homeostasis [50,51], may engage in crosstalk with PGE2 signaling to influence oxidative stress responses and cellular protection. Given the emerging role of LCN2 in redox regulation, NRF2 signaling may represent an important modulatory arm of this pathway. Dissecting the contributions and interactions of these pathways will require targeted pharmacologic and genetic experimental approaches.

Beyond PGE2, other 15-PGDH substrates such as resolvin D1 (RvD1) and lipoxin A4 (LXA4) may act synergistically within a broader homeostatic autocoid network to contribute to the observed neuroprotective effects. RvD1 levels are reduced in PD patients and in human α-synuclein overexpressing rats, and its chronic administration ameliorates neuroinflammation and motor deficits in rodent PD models [52]. Similarly, LXA4 suppresses microglial activation in both *in vitro* and *in vivo* models of neuroinflammation [53,54]. Although these pathways are not captured in BV-2 cells, we hypothesize that 15-PGDH inhibition targets a broad regulatory node and may provide an effective strategy for simultaneously engaging the therapeutic activity of multiple specialized pro-resolving mediators, including LXA4 and RvD1.

Importantly, 15-PGDH inhibition significantly reduced markers of oxidative stress, including 4-HNE and 3-NT, across three PD animal models. *In vitro*, PGE2 suppressed both NOX2 expression and superoxide production in LPS-stimulated microglia. This is consistent with a previous report that 15-PGDH inhibition in a stroke model suppressed lipid peroxidation through transcriptional upregulation of glutathione peroxidase 4 (GPX4) via the PGE2/EP4 axis [55].

Further investigation into the regulatory mechanisms governing *Hpgd* expression will be important for understanding the upstream processes that drive 15-PGDH elevation in PD. While *in vitro* experiments have demonstrated that PGE2 can reduce LCN2 and NOX2 expression, *in vivo* studies will be necessary to fully interrogate the interactions among these signaling pathways. Additional experiments, including *in vivo* EP4 knockout and inhibition, NOX2 silencing, and LCN2 neutralization, will further validate these findings and clarify their mechanistic and therapeutic implications. In addition, further validation in additional human patient samples will be important in future studies.

In conclusion, our findings establish 15-PGDH as a robust therapeutic target in PD. 15-PGDH levels are elevated in the brains of PD mice and humans, and both genetic and pharmacologic inhibition produces marked therapeutic efficacy in mouse PD models. Previous work from our team demonstrated high CNS penetration of SW033291, with sustained drug levels in both brain and plasma sustained for up to 6 h, and, as shown in the present study (Fig. S1A), near-complete ablation of 15-PGDH enzyme activity in the brain. The clinical safety of 15-PGDH inhibition is supported by the absence of toxicity in a recent human phase 1 trial of the 15-PGDH inhibitor MF-300 [56], as well as by findings from humans with biallelic inactivating mutations of 15-PGDH, in whom the only consistently observed phenotype is congenital digital clubbing [57]. Encouragingly, both pharmaceutical and biotechnology companies have initiated development of 15-PGDH inhibitors for peripheral indications, and inhibitor MF-300 has already completed human phase 1 trials. Our results now provide the rationale to repurpose such agents for the treatment of PD.

## Funding

M − KS was supported by the New Faculty Startup Fund (370C-20220110), Creative-Pioneering Researchers Program (370C-20230108), and a research grant (370C-20240120) from Seoul National University. M − KS also acknowledges support from the National Research Foundation of Korea (RS-2023-00209597, RS-2024-00352229, RS-2024-00466703, RS-2025-25444385/NTIS: 2710092625, RS-2026-25475429), Seoul R&BD program (BT240041) and donors of Alzheimer's Disease Research, a program of BrightFocus Foundation (A2019551F). Y.P.K. was supported by the New Faculty Startup Fund and Creative-Pioneering Researcher’s Program (370C-20230107) and a research grant (370C-20240120) from Seoul National University and National Research Foundation of Korea (NRF-2022M3A9I2017587, NRF-2022R1C1C1003619). A.A.P. and S.D.M. were supported by NIH/NIGMS RM1 GM142002. A.A.P. was also supported by The Valour Foundation, the Wick Foundation, Department of Veterans Affairs Merit Award I01BX005976, and as the Rebecca E. Barchas, MD, Professor in Translational Psychiatry of Case Western Reserve University and the Morley-Mather Chair in Neuropsychiatry of University Hospitals of Cleveland Medical Center. A.A.P. and B.D.P. were supported by the American Heart Association and Paul Allen Foundation Initiative in Brain Health and Cognitive Impairment (19PABH134580006) and by NIH/NIA 1R01AG071512. A.A.P. also acknowledges support from NIH/NIA RO1AGs066707, NIH/NIA 1 U01 AG073323, the Louis Stokes VA Medical Center resources and facilities, the Lincoln Neurotherapeutics Research Fund, the Leonard Krieger Fund of the Cleveland Foundation, and an anonymous donor. B.D.P. acknowledges support from NIH/NIDA P50 DA044123, NIH/NIA 1R21AG073684-01 and RO1AG071512, and funding from the Solve-ME Foundation and the Catalyst Award fromJohns Hopkins University. S.D.M. acknowledges support from the Markowitz-Ingalls Chair in Cancer Genetics at Case Western Reserve University, from the Case Comprehensive Cancer Center, and from philanthropic donors. Y.K. was supported by NIH/NIA F99 AG083111. E.V–R. was supported by Department of Defense Peer-Reviewed Alzheimer's Research Program (PRARP) Award AZ210092 (W81XWH-22-1-0129). S.J.M. was supported by Basic Science Research Program through the National Research Foundation of Korea (NRF) funded by the Ministry of Education (2021R1A6A1A03040260). We thank Dr. Hyang-Sook Hoe (Department of Neural Development and Disease, Korea Brain Research Institute, Republic of Korea) for providing the BV2 cell line. We also acknowledge BioRender, a service we used to design our schematic figures and graphical abstract.

## CRediT authorship contribution statement

**Young-Kwang Kim:** Formal analysis, Investigation, Writing – review & editing. **Yun Jae Cha:** Formal analysis, Investigation, Writing – review & editing. **Se Eun Park:** Formal analysis, Investigation, Writing – review & editing. **Hee Kyung Kim:** Investigation, Writing – review & editing. **Chaesun Kwon:** Investigation, Writing – review & editing. **Geonmo Kim:** Investigation, Writing – review & editing. **Yoonah R. Oh:** Investigation, Writing – review & editing. **Edwin Vázquez-Rosa:** Formal analysis, Investigation, Writing – review & editing. **Ujjwal Dahiya:** Formal analysis, Investigation, Writing – review & editing. **Helen Moinova:** Formal analysis, Investigation, Writing – review & editing. **Yeojung Koh:** Formal analysis, Investigation, Writing – review & editing. **Farrah Gao:** Investigation, Writing – review & editing. **Sunil Jamuna Tripathi:** Formal analysis, Investigation, Writing – review & editing. **Suwarna Chakraborty:** Formal analysis, Investigation, Writing – review & editing. **Dong-Gyu Jo:** Investigation, Writing – review & editing. **Minseo Woo:** Investigation, Writing – review & editing. **Hyejin Park:** Investigation, Writing – review & editing. **Seung-Jae Myung:** Investigation, Writing – review & editing. **Jiwon Cheon:** Investigation, Writing – review & editing. **Yunjong Lee:** Investigation, Writing – review & editing. **Xin Qi:** Formal analysis, Investigation, Writing – review & editing. **Bindu D. Paul:** Formal analysis, Funding acquisition, Investigation, Writing – review & editing. **Stephen Fink:** Investigation, Writing – review & editing. **Lakshmi Kasturi:** Formal analysis, Investigation, Writing – review & editing. **James Lutterbaugh:** Formal analysis, Investigation, Writing – review & editing. **Sanford D. Markowitz:** Conceptualization, Formal analysis, Funding acquisition, Supervision, Writing – original draft, Writing – review & editing. **Andrew A. Pieper:** Conceptualization, Formal analysis, Funding acquisition, Supervision, Writing – original draft, Writing – review & editing. **Yun Pyo Kang:** Conceptualization, Formal analysis, Funding acquisition, Supervision, Writing – original draft, Writing – review & editing. **Min-Kyoo Shin:** Conceptualization, Formal analysis, Funding acquisition, Supervision, Writing – original draft, Writing – review & editing.

## Declaration of competing interest

The authors declare the following financial interests/personal relationships which may be considered as potential competing interests:Yeojung Koh has patent issued to n/a. Edwin Vazquez-Rosa has patent issued to n/a. Sanford D. Markowitz has patent issued to n/a. Andrew A. Pieper has patent issued to n/a. Min-Kyoo Shin has patent issued to n/a. If there are other authors, they declare that they have no known competing financial interests or personal relationships that could have appeared to influence the work reported in this paper.

## Data Availability

Data will be made available on request.
