# Supplementary material for: Inhibiting 15-PGDH restores redox homeostasis and confers neuroprotection in Parkinson's disease
Source: Redox Biol. 2026 Jun 30;95:104285. doi: 10.1016/j.redox.2026.104285 (PMC13351393; doi:10.1016/j.redox.2026.104285)
Supplement: Multimedia component 1 [file mmc1.docx]

**Supplemental Materials**

**Methods**

*MPTP administration*

To examine the effect of pharmacological inhibition of 15-PGDH on the MPTP-induced PD model, seven-week-old male C57BL/6J mice were acclimated for one week and randomly divided into four groups: vehicle-treated (n = 10), MPTP-treated (n = 10), MPTP+ 1 mg/kg of SW033291-treated (n = 11), and MPTP + 10 mg/kg of SW033291-treated (n = 10). Animals were intraperitoneally administered 0.5 or 5 mg/kg of the 15-PGDH inhibitor SW033291 twice daily (in the early morning and late afternoon) for two days, after which they were administered MPTP (30 mg/kg) (Sigma-Aldrich, M0896) for 7 consecutive days, with twice daily SW033291 treatment also continuing. After the final MPTP injection, vehicle or SW033291 was administered for three additional days. To examine the effect of genetic inhibition of 15-PGDH, eight-week-old *Hpgd*^+/+^ and *Hpgd*^+/-^ mice were randomly divided into three groups: *Hpgd*^+/+^ with vehicle-treatment (male = 15, female = 7), *Hpgd*^+/+^ with MPTP-treatment (male = 9, female = 6), and *Hpgd*^+/-^ with MPTP-treatment (male = 12, female = 7). MPTP (30 mg/kg) was administered for 7 consecutive days. All behavior tests were performed two hours after the final MPTP injection.

*Intranigral LPS administration*

For intranigral LPS administration, mice were anesthetized with 2.5% isoflurane (in 100% oxygen at a flow rate of 0.5 L/min) and positioned in a stereotaxic frame with non-rupture ear bars. To ensure continuous anesthesia, the frame was equipped with a nose cone connected to a calibrated vaporizer. LPS (3 μg, 1 μg/μL) was bilaterally injected into the substantia nigra using a Hamilton syringe (Hamilton, 7635-01) connected to an electronic infusion pump (HUAYON, 20-0100) at a rate of 0.5 μL/min for 6 min per hemisphere. An equivalent volume of distilled water (D.W.) was administered as a control. Coordinates to bregma for injection were −3.1 mm (AP), ±1.2 mm (ML), and −4.4 mm (DV). To minimize potential reflux, the needle was maintained in position for 10 min following each injection before being gradually withdrawn.

*Stereotaxic injection of α-syn PFF and AAV-human α-syn*

Male C57BL/6J mice (2 months old) were purchased from JA BIO Animal Company (Suwon, Korea). Mice were anesthetized with 2.5% isoflurane (100% O₂; 0.5 L/min) and mounted in a stereotaxic frame equipped with non-rupture ear bars. Anesthesia was maintained throughout the procedure via a calibrated vaporizer. For viral and fibril injections, AAV-eGFP or AAV-αSyn (2.0 × 10^12 GC/mL; 1 μL/side) were bilaterally injected into the SN (AP −3.4 mm, ML ±1.2 mm, DV −4.3 mm), and PBS or α-synuclein preformed fibrils (αSyn PFF; 5 mg/mL; 2 μL/side) were injected into the ventral tegmental area (VTA; AP −3.4 mm, ML ±0.5 mm, DV −4.3 mm).

*Stereotaxic injection of α-syn PFF*

a-syn PFFs were prepared as previously described^1^ by agitating (1,000 rpm at 37°C) for 7 days and sonicating for 30 s (0.5 sec pulse on/off) at 10% amplitude (Branson Digital Sonifier). Two to three-month-old WT mice were anesthetized with a mixture of avertin (240 mg/kg) and fixed in a stereotaxic instrument. PBS or α-syn PFF (5 μg) were bilaterally injected into striatum (2 μl per hemisphere at 0.4 μl/min): anteroposterior (AP) = +0.2 mm, mediolateral (ML) = + 2.0 mm, dorsoventral (DV) = −2.8 mm from bregma. After injection, the needle was maintained in position for an additional 3 min for a complete absorption. After surgery, animals were monitored the following day. Tissue samples were collected 6 months after injection and frozen at -80°C.

*RT-qPCR*

Total RNA was purified from brain tissue and cells using the TRI Reagent (Molecular Research Center, TR118) according to the manufacturer’s instructions. RNA 1 µg was treated with DNase I (Enzynomics, M0595) and reverse transcribed with M-MLV Reverse Transcriptase and Random Hexamers (Enzynomics, RT001S). Quantitative PCR was performed using BioFACT Real time PCR Master Mix (BIOFACT, DQ383-40h) including SYBR Green in mixture on a QuantStudio^TM^ 5 Real-Time PCR System (Thermo Fisher Scientific, A28138). Expression levels of *hpgd* (human forward: GGT TCC ACT GAT AAC AGA AAC CA, human reverse: TTT GGT CAA TAA TGC TGG AGT G, mouse forward: GTG AAC GGC AAA GTG GCT CT, mouse reverse: TCC AAT CCA CCA ATG CTA CCT), *lcn2* (forward: CCC CAT CTC TGC TCA CTG TC, reverse: TTT TTC TGG ACC GCA TTG), and *cybb* (forward: TGG CGA TCT CAG CAA AAG GTG G, reverse: GTA CTG TCC CAC CTC CAT CTT G) were determined by the ΔΔCt method and normalized to the expression of *gapdh* (human forward: GTC TCC TCT GAC TTC AAC AGC G, human reverse: ACC ACC CTG TTG CTG TAG CCA A, mouse forward: CAT CAC TGC CAC CCA GAA GAC TG, mouse reverse: ATG CCA GTG AGC TTC CCG TTC AG).

*Western blotting*

Mouse brain tissues and cells were homogenized in RIPA buffer (Thermo Fisher Scientific, 89901) containing protease inhibitor (Thermo Fisher Scientific, 78429) and phosphatase inhibitor (Merck, 04 906 837 001). Lysates were centrifuged at 14,000 rpm at 4°C for 30 min, after which supernatants were collected. Bicinchoninic acid assay (Thermo Fisher Scientific, A53225) was used for measuring protein concentration. Proteins were heated in Laemmli Sample Buffer (Bio-Rad Laboratories, Inc., #1610737) with beta-mercaptoethanol (Bio-Rad Laboratories, Inc., #1610710) for 5 min and then resolved in sodium dodecyl sulfate-polyacrylamide gel electrophoresis. Proteins were transferred onto 0.45 µm polyvinylidene fluoride membranes (Sigma-Aldrich, IPVH00010) with the Trans-Blot Turbo system (Bio-Rad Laboratories, Inc., 1704150). Membranes were blocked with 5% nonfat dry milk (MB Cell, MB-S1667) or bovine serum albumin (Bovogen Biologicals, BSAS 0.1) in tris-buffered saline-tween 20 (TBST) for 1 h at room temperature.

After blocking, membranes were cut according to the molecular weights of the proteins of interest and incubated separately with the appropriate primary antibodies against the target proteins and their corresponding loading controls at 4°C overnight. The following antibodies were used to probe target proteins: rabbit anti-15-PGDH (1:500, Novus Biologicals, NB200-179), rabbit anti-GAPDH (1:10,000, EMD Millipore Corp, MAB374), rabbit anti-tyrosine hydroxylase (1:2000, Merck Millipore, AB-152), mouse anti-β-actin (1:2000, Cell Signaling Technology, 3700S), goat anti-LCN2 (1:2000, R&D Systems, AF1857), and rabbit anti-NOX2/gp91phox (1:1000, Abcam, ab310337). After primary antibody incubation, membranes were rinsed with TBST (3 x 5 min) and subsequently incubated with horseradish peroxidase-conjugated secondary antibodies. Immobilon Western Chemiluminescent HRP Substrate (Merck, WBKLS0050) was used to detect the band by a Biomolecular Imaging System (Cytiva, ImageQuant 800 F). Densitometry quantification of western blot signal was conducted by ImageJ version 1.53 software (National Institutes of Health, Bethesda, MD). Target protein levels were normalized to the corresponding loading control signals.

*15-PGDH enzyme activity*

For pharmacologic studies, mice were euthanized 30 minutes after the last dose of vehicle or (+)-SW033291 injection and brain tissues were excised. Tissues were further rinsed in ice-cold PBS and snap-frozen in liquid nitrogen. Frozen samples were pulverized over liquid nitrogen. The powder was transferred to an Eppendorf tube with 500 μL of cold lysis buffer (50 mM Tris-HCl, pH 7.5, 0.1 mM DTT, 0.1 mM EDTA) and then homogenized using Kimble Kontes Pellet Pestle Cordless Motor (Fisher, K749540-0000) with Kimble Kontes blue pellet pestles (Fisher, K749521-1590) for ~ 30s on ice. The suspension was centrifuged for 10 min at 12,000 rpm. Enzyme activity was measured by transfer of tritium from a tritiated PGE2 substrate to glutamate by coupling 15-PGDH to glutamate dehydrogenase in a 1 hour reaction with 1mM NAD+, 5 mM NH4Cl, 1 mM α-keto-glutarate (Sigma-Aldrich, #75890), and 16 U of bovine liver glutamic dehydrogenase (Sigma-Aldrich, #G-2501), as described previously^2^. Enzymatic activity was normalized to protein concentration measured by BCA assay (Thermo Scientific, #23225) and expressed as CPM/Hour/mg protein.

*Behavioral analysis*

All behavior experiments were conducted 1h after acclimation in the behavior room. The rotarod test was used to assess motor coordination. The speed of the rod was accelerated gradually from 4 to 20 rpm over a period of 120 s. This training process was repeated three times to habituate all mice to walk on the rod. Following the training, mice were tested for 3 consecutive trials on the rod for 120 s, with the speed of the rod accelerated gradually from 4 to 40 rpm over a period of 120 s.

The clasping test was rated from 0 to 2 based on severity: 0 = hindlimb splayed outward and away from the abdomen; 1 = one hindlimb retracted towards the abdomen; 2= both hindlimb retracted toward the abdomen. The mice were lifted by the base of the tail and their behaviors were observed and recorded for 30 seconds. This procedure was repeated three times during the test.

In the pole test, mice were positioned facing upward on the top of a 50 cm vertical, 1 cm diameter wooden pole. Bedding material was used to cover the base of the pole to protect the mice from potential injuries. All mice underwent three trials to measure the time to go down and to turn heads downward.

Clasping and rotarod tests were performed 2 h after the final MPTP injection and pole test was conducted 24 h after the final MPTP injection. All behavior tests and analyses were conducted blind to the treatment group.

*Immunohistochemistry*

Mice were anesthetized and euthanized by transcardial perfusion with cold 1 x phosphate-buffered saline (PBS) followed by 4% paraformaldehyde in PBS at pH 7.4. Brains were carefully removed and post-fixed in 4% paraformaldehyde for 24 h at 4°C, immersed in 30% sucrose in PBS for 72 h at 4°C, and then frozen using an Optimal Cutting Temperature (OCT) compound and stored in -80°C. The samples were coronally sectioned at 30 µm thickness using a cryotome (CM3050, Leica) and sections were stored in cryoprotective solution (100 mM glycerol, 150 mM ethylene glycol, 250 mM PBS) at - 20°C. For immunohistochemistry, sections were rinsed in 1 x PBS (5 min, three times) and incubated with 0.2 % Triton X-100 in PBS for 15 min. After washing with 1 x PBS (5 min, three times), sections were incubated in blocking buffer (5 % normal horse serum and 0.2 % Triton X-100 in PBS) for 1 h at RT. The sections were incubated overnight at 4°C with primary antibodies (TH: 1:1000, Iba1: 1:1000, GFAP: 1:1000, 4-HNE: 1:500, 3-NT: 1:500). After washing in PBS (5 min, three times), sections were incubated with Alexa Fluor 555 goat anti-rabbit (Invitrogen, A21428) or Alexa Fluor 555 goat anti-mouse (Invitrogen, A21422) secondary antibodies (TH: 1:1000, Iba1: 1:1000, GFAP: 1:1000, 4-HNE: 1:300, 3-NT: 1:300) at room temperature for 2 h in darkness. Sections were rinsed with 1 x PBS (5 min, three times) and mounted on slides and coverslipped with antifade mounting solution.

*Quantification of immunohistochemistry*

Images were captured using a confocal microscope (TCS SP8, Leica) and slide scanner (Vectra, PerkinElmer). ImageJ version 1.53 software (NIH, Bethesda, MD) was used to analyze fluorescent images. The operator performing quantification was blinded to the condition and treatment.

*Measurement of MAO-B activity*

MAO-B activity was measured by using a commercially available kit (Thermo Fisher Scientific, A12214), according to the manufacturer’s instructions. Samples were pre-incubated for 30 min at room temperature with the specific MAO-A inhibitor, clorgyline (1 μM), and then put into individual wells of a 96-well microplate. The fluorometric assay was initiated by adding 100 μl of a reaction mixture that contained Amplex Red reagent (400 μM), horseradish peroxidase (HRP, 2 U/ml), and the MAO-B substrate benzylamine (2 mM). Plates were incubated for 30 min at room temperature and fluorescence was measured at excitation and emission wavelengths of 545 nm and 590 nm, respectively, using a fluorescence microplate reader (Molecular Devices, SpectraMAX M5). Hydrogen peroxide (10 μM) was used as a positive control and reaction buffer was used as a negative control.

*Bulk RNA sequencing*

Following dissection of the substantia nigra from the whole brain, total RNA was extracted using the XENOPURE™ PF-Total RNA Purification Kit (XENOHELIX, 9366RTR) according to the manufacturer’s instructions. RNA samples were sent to NovogeneAIT Genomics Singapore Pte. Ltd. (Singapore) for bulk RNA-seq using the NovaSeq 6000 platform. Differentially expressed genes (DEGs) were identified using the DESeq2 package (v 1.44.0) in R and p-values were adjusted using Benjamini-Hochberg (BH) method. For Gene Ontology (GO) enrichment analysis in the Biological Process (BP) category, genes with p < 0.05 and |log2 fold change| > 0 were selected. The GO analysis was conducted using the clusterProfiler package (v 4.12.6), and the results were visualized using enrichplot (v 1.24.4) and ggplot2 (v 3.5.1). A heatmap was generated using the ComplexHeatmap (v 2.20.0) to illustrate expression patterns across samples. All analyses were performed in RStudio.

*Superoxide measurement*

BV2 cells (AcceGen Biotechnology, ABC-TC212S) were seeded into 96-well white, clear-bottom microplates at a density of 20,000 cells/well in 100 µL of standard growth media. Following plating, cells were pre-treated with 5 µM prostaglandin E2 (PGE2). Vehicle control wells received an equivalent volume of dimethyl sulfoxide (DMSO). The plates were maintained in a humidified incubator at 37°C with 5% CO2 for 24 hours. Following 24-hour substrate pre-treatment with 15-PGDH substrates, cells were stimulated with 0.1 µg/mL lipopolysaccharide (LPS).  Baseline control wells received an equivalent volume of sterile water. The plates were then returned to the 37°C incubator for an additional 24-hour stimulation period. After the 24-hour LPS treatment, culture media was aspirated and cells were washed with 1 x Hank's Balanced Salt Solution (HBSS) and subsequently submerged in 100 µL of fresh HBSS. To quantify superoxide production, 100 µL of a 2X L-012 (400 uM) chemiluminescent probe detection mixture (Wako chemicals, catalogue no. 120-04891) (prepared in water) was added to the wells, resulting in a final working concentration of 200 µM L-012. Luminescence was recorded immediately upon addition of the L-012 detection buffer using a Perkin Elmer microplate reader. To capture the trajectory of the oxidative burst, kinetic measurements were acquired every 2 minutes for a total duration of 60 minutes at 37°C. Plateau phase values recorded at 30 minutes were used for comparison across conditions. Luminescence values were normalized for well confluence assessed by incucyte estimation performed at the completion of the assays. To confirm NADPH oxidase specificity of the L-012 luminescence assays, control cells were treated for 1 hour before the assay with 10 uM of GSK2795039 NOX2 inhibitor added in culture media and the detection buffer.

*Electron microscopy*

Mice were anesthetized and euthanized by transcardial perfusion with a fixative solution (1/4 strength Karnovsky’s fixative) at a flow rate of 10 ml/min for 10 min. The brains were carefully removed and then sliced into thin slices (4 mm x 4 mm x 2 mm). Tissues were fixed with 2.5% glutaraldehyde, 2% paraformaldehyde in 0.1 M HEPES buffer, pH 7.4 for 2h at room temperature. The specimens were thoroughly rinsed in 0.1 M phosphate buffer, pH7.4, then postfixed for 2 h in an unbuffered 1:1 mixture of 2% osmium tetroxide and 3% potassium ferricyanide. After rinsing with distilled water, the specimens were soaked overnight in an acidified solution of 0.25% uranyl acetate. After another rinse in distilled water, they were dehydrated in ascending concentrations of ethanol, passed through propylene oxide, and embedded in EMbed 812 resin mixture (Electron Microscopy Sciences, PA). Thin sections (70 nm) were cut on an RMC MT6000-XL ultramicrotome and mounted on Gilder square 300 mesh nickel grids (Electron Microscopy Sciences, PA). Thin sections were sequentially stained with acidified uranyl acetate followed by a modification of Sato’s triple lead stain and examined in a FEI Tecnai Spirit (T12) with a Gatan US4000 4kx4k CCD. The operator conducting quantification was blinded to condition and treatment.

*Tissue sample preparation for prostaglandin extraction*

For pharmacologic studies, mice were euthanized 3 hours after the last dose of vehicle or (+)-SW033291 injection and brain tissues were excised. PGE_2_-d_4_ was purchased from Cayman Chemicals (314010). Extraction solvent was prepared by mixing PGE_2_-d_4_ to a final concentration of 10 nM in a 1:1 v/v acetone/water solution containing 0.005% butylated hydroxytoluene (BHT). The prostaglandin extraction procedure was slightly modified from the previous method^3^. Snap-frozen substantia nigra and striatum were pulverized using a multisample biopulverizer (BioSpec) on dry ice. Pulverized brain tissues were rapidly weighed on a scale and transferred into EP tube. Two hundred microliters of extraction solvent were added and tissue was homogenized via ultrasonication using a Bioruptor BMS (30 sec on, 30 sec off cycle, 5 min). Two hundred microliters of extraction buffer were further added into the homogenate, vortexed, and centrifuged for 20 min at 17,000 rpm at 4°C. Three hundred microliters of supernatant were transferred to a new EP tube followed by addition of 300 µL of hexane. The extract was shaken for 15 min using Vortex Mixer at 4°C, followed by centrifugation at 17,000 rpm for 20 min at 4°C. After the aqueous layer solidified at -80°C, the hexane layer was removed. After thawing the aqueous layer on ice, 37.5 µL of 1 M formic acid was added and vortexed. For the second extraction, 300 µL of chloroform was added to the aqueous layer. Samples were then shaken for 15 min at 4°C. After centrifugation, the lower chloroform layer was transferred to a new EP tube and evaporated using a Speedvac at 30°C. The dried extract was reconstituted in 20 µL ACN/water (3:7) containing 0.1% acetic acid.

*LC-MS/MS-based prostaglandin analysis*

LC-MS/MS analysis was performed using an Agilent 6460 Triple Quad Mass spectrometer coupled with an Agilent 1290 Infinity Series LC system (Agilent Technologies). A Brownlee SPP C18 (2.1 x 75mm, 2.7 µm) Column (Perkinelmer) was used with column temperature at 30°C. Mobile phase A consisted of 0.1% acetic acid H_2_O and mobile phase B consisted of 0.1% acetic acid in ACN with a flow rate of 0.3 mL/min. The gradient conditions for the solvent were 0 min, 20% B; 0-3.5 min, 45% B; 3.5-7 min, 60% B; 7-9 min, 95% B; 9-10 min, 95% B; 10-10.1 min, 20% B; 10.1-12 min, 20% B. The injection volume of the sample was 5 µL. Multiple reaction monitoring (MRM) of LC-MS/MS was used to quantify prostaglandin levels with the following transitions: PGE_2_ (Q1=351.1, Q2=271.2, CE=16), and PGE_2_-d_4_ (Q1=355.1, Q2=275.1, CE=16). Peak integration and quantification were performed using MassHunter software (Agilent) with the isotope dilution method.

*Quantification and statistical analysis*

Statistical analyses were performed using GraphPad Prism, version 10.0.3 (GraphPad Software, Inc.). Student’s t-test was used to demonstrate statistical between two groups, and One-way ANOVA with Tukey’s post hoc test was used when appropriate. Values are presented as mean ± SEM, and individual data points represent individual samples or animals. Details of statistical analysis can be found in the figure legends.

**Supplemental References**

1. Kam, T.I., Mao, X., Park, H., Chou, S.C., Karuppagounder, S.S., Umanah, G.E., Yun, S.P., Brahmachari, S., Panicker, N., Chen, R., et al. (2018). Poly(ADP-ribose) drives pathologic alpha-synuclein neurodegeneration in Parkinson's disease. Science *362*. 10.1126/science.aat8407.

2. Tong, M., and Tai, H.H. (2004). Synergistic induction of the nicotinamide adenine dinucleotide-linked 15-hydroxyprostaglandin dehydrogenase by an androgen and interleukin-6 or forskolin in human prostate cancer cells. Endocrinology *145*, 2141–2147. 10.1210/en.2003-1229.

3. Prasain, J.K., Hoang, H.D., Edmonds, J.W., and Miller, M.A. (2013). Prostaglandin extraction and analysis in Caenorhabditis elegans. J Vis Exp. 10.3791/50447.


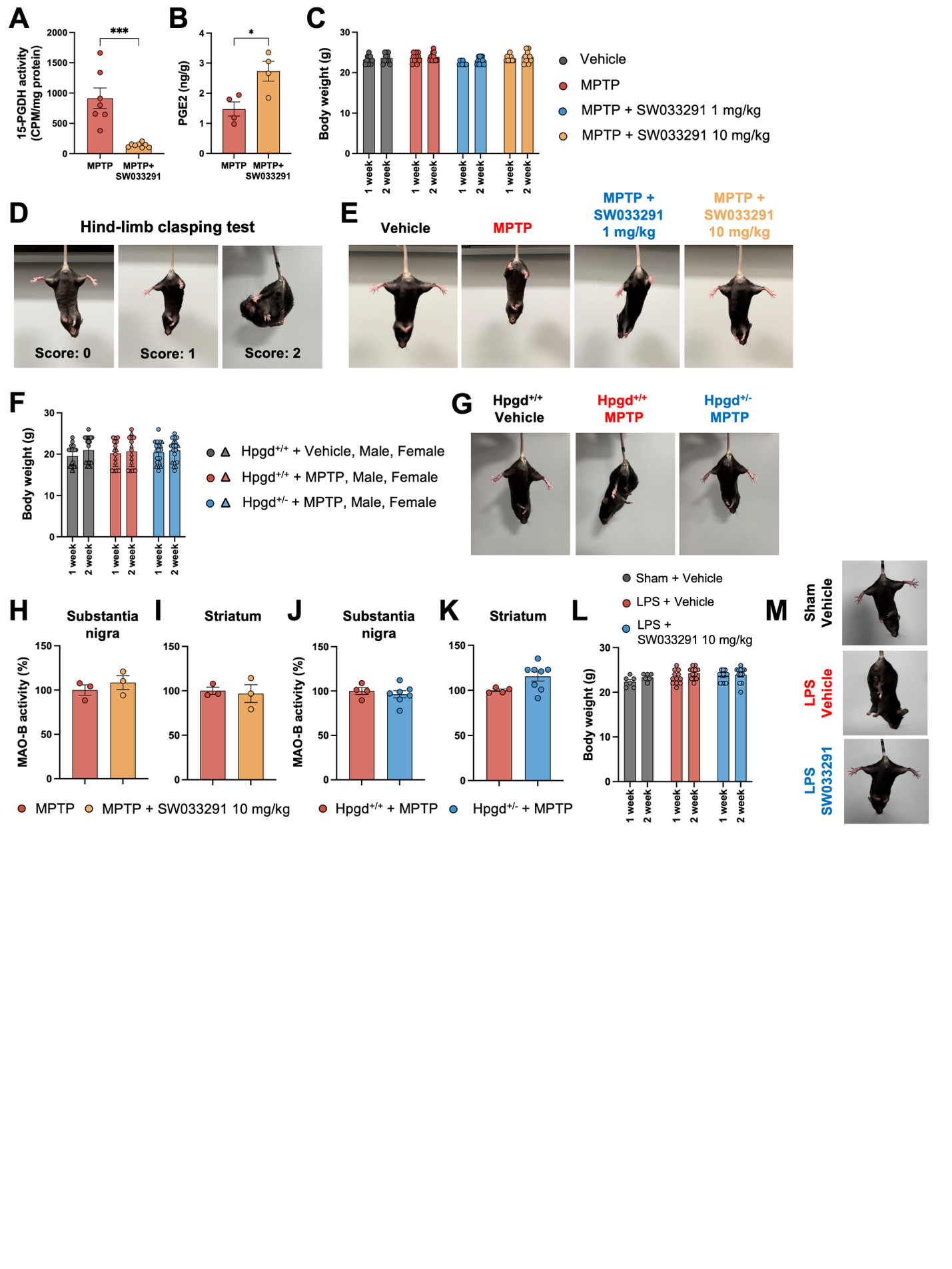


**Fig. S1. 15-PGDH inhibition elevates PGE2 levels without affecting body weight and MAO-B activity**

(A) 15-PGDH activity is significantly reduced by SW033291 in MPTP-treated mice (n=7-8 per group, *** p < 0.001, unpaired t-test).

(B) SW033291 elevated brain PGE2 levels in MPTP-treated mice (n=4 per group, * p < 0.05, unpaired t-test).

(C) Body weight was not altered by treatment with MPTP and SW033291 (n=10-11 per group).

(D) Representative posture and scoring in the hind-limb clasping test.

(E) Representative pictures of the hind-limb clasping test shown in Figure 2C.

(F) Body weight showed no significant differences between *Hpgd* heterozygous mice and their wild-type littermates treated with vehicle or MPTP (n=10-14 per group, males and females are shown as circles and triangles, respectively).

(G) Representative pictures of the hind-limb clasping test shown in Figure 2G.

(H) SW033291 did not affect monoamine oxidase B activity in the substantia nigra of MPTP-treated mice (n=3 per group).

(I) SW033291 did not affect monoamine oxidase B activity in the striatum of MPTP-treated mice (n=3 per group).

(J) Monoamine oxidase B activity was similar between MPTP-treated *Hpgd* heterozygous mice and their wild-type littermates in the substantia nigra (n=4-7 per group).

(K) Monoamine oxidase B activity was similar between MPTP-treated *Hpgd* heterozygous mice and their wild-type littermates in the striatum (n=4-8 per group).

(L) Body weight was not affected by treatment with LPS and SW033291 (n=6-12 per group).

(M) Representative pictures of the hind-limb clasping test shown in Figure 2K.


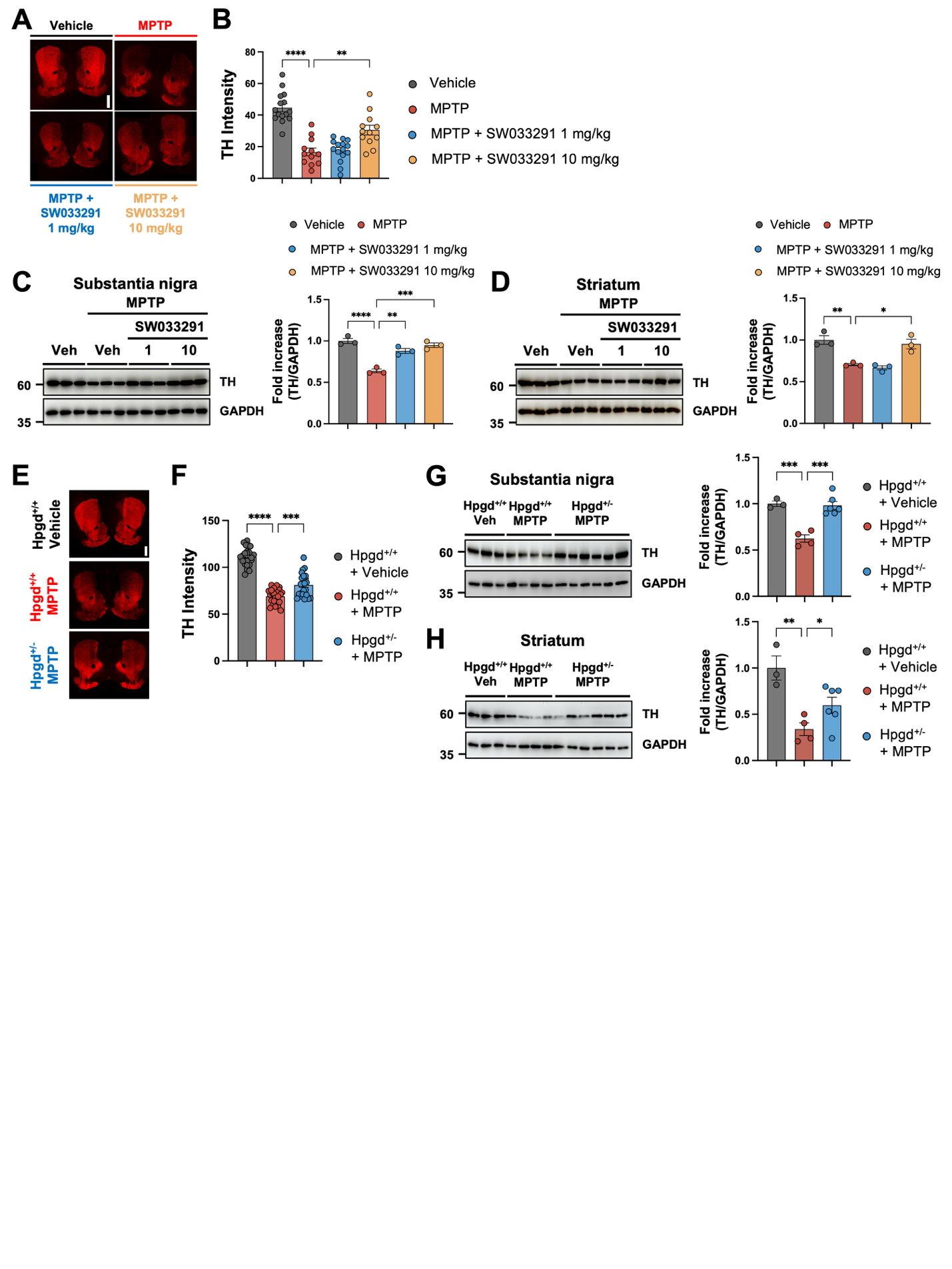


**Fig. S2. 15-PGDH inhibition protects against dopaminergic neuronal loss in MPTP-induced PD models**

(A) Representative TH-stained images of the striatum of mice treated with vehicle or MPTP in the absence or presence of SW033291 (scale bar = 800 µm).

(B) Quantification of TH fluorescence signal shows that SW033291 treatment increased the TH intensity in the striatum of mice exposed to MPTP (n=4 per group, ** p < 0.01, **** p < 0.0001, one-way ANOVA and Tukey’s post hoc analysis).

(C) Western blot and its quantification show that SW033291 dose-dependently increased TH levels in the substantia nigra of mice exposed to MPTP (n=3 per group, ** p < 0.01, *** p < 0.001, **** p < 0.0001, one-way ANOVA and Tukey’s post hoc analysis).

(D) Western blot and its quantification show that SW033291 increased TH levels in the striatum of mice exposed to MPTP (n=3 per group, * p < 0.05, ** p < 0.01, one-way ANOVA and Tukey’s post hoc analysis).

(E) Representative TH-stained images of the striatum from *Hpgd* heterozygous mice and their wild-type littermates treated with vehicle or MPTP (scale bar = 800 µm).

(F) Quantification of TH fluorescence signal shows that *Hpgd* heterozygous mice have higher TH intensity than their wild-type littermates treated with MPTP (n=4 per group, *** p < 0.001, **** p < 0.0001, one-way ANOVA and Tukey’s post hoc analysis).

(G) Western blot and its quantification show that *Hpgd* heterozygous mice have increased TH levels in the substantia nigra compared to their wild-type littermates exposed to MPTP (n=3-6 per group, *** p < 0.001, one-way ANOVA and Tukey’s post hoc analysis).

(H) Western blot and its quantification show that *Hpgd* heterozygous mice have increased TH levels in the striatum compared to their wild-type littermates treated with MPTP (n=3-6 per group, * p < 0.05, ** p < 0.01, one-way ANOVA and Tukey’s post hoc analysis).

**
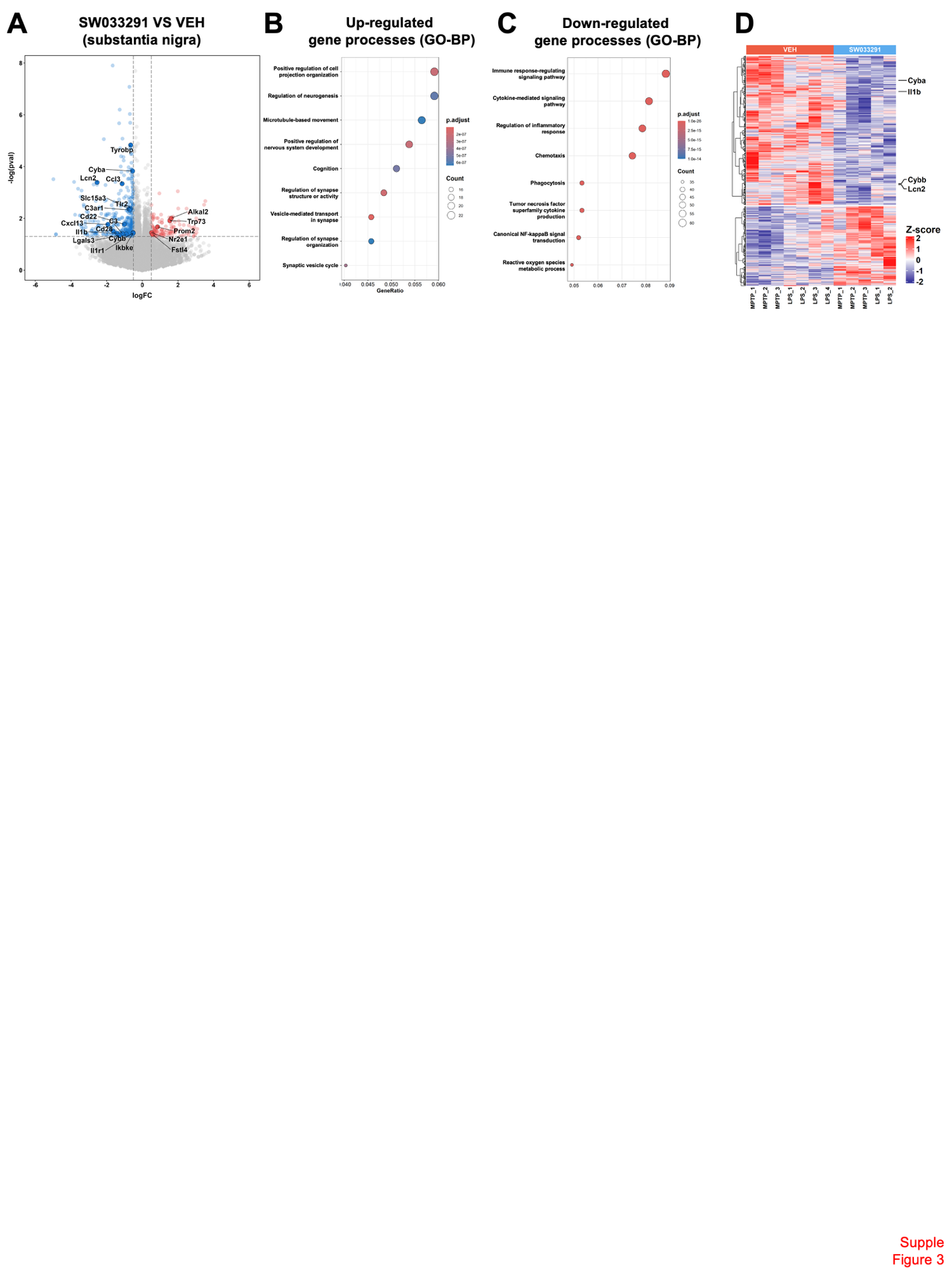
**

**Fig. S3. Inhibition of 15-PGDH markedly suppresses immune and inflammatory responses and reactive oxygen species metabolism in bulk RNA-seq analysis**

(A) Volcano plot illustrating differentially expressed genes (DEGs) identified from bulk RNA-seq analysis, comparing the SW033291-treated group (n=5) versus the vehicle-treated group (n=7) {MPTP + SW033291 (n=3) and LPS + SW033291 (n=2) versus MPTP + vehicle (n=3) and LPS + vehicle (n=4)}. Red and blue dots represent upregulated and downregulated genes, respectively, with (|logFC| > 0.5, and p-value < 0.05). Dots with black outlines highlight notable genes.

(B) Gene Ontology (GO) enrichment analysis of Biological Processes associated with upregulated genes having p-value < 0.05. Depicted are the top 9 most significant results spanning q=1.66e-4 to q=2.13e-4.

(C) GO enrichment analysis for Biological Processes associated with downregulated genes having p-value < 0.05. Depicted are selected representative pathways from the top 40 most significant results spanning q=1.65e-23 to q=3.56e-14.

(D) Heatmap depicting gene expression patterns across all groups.

**
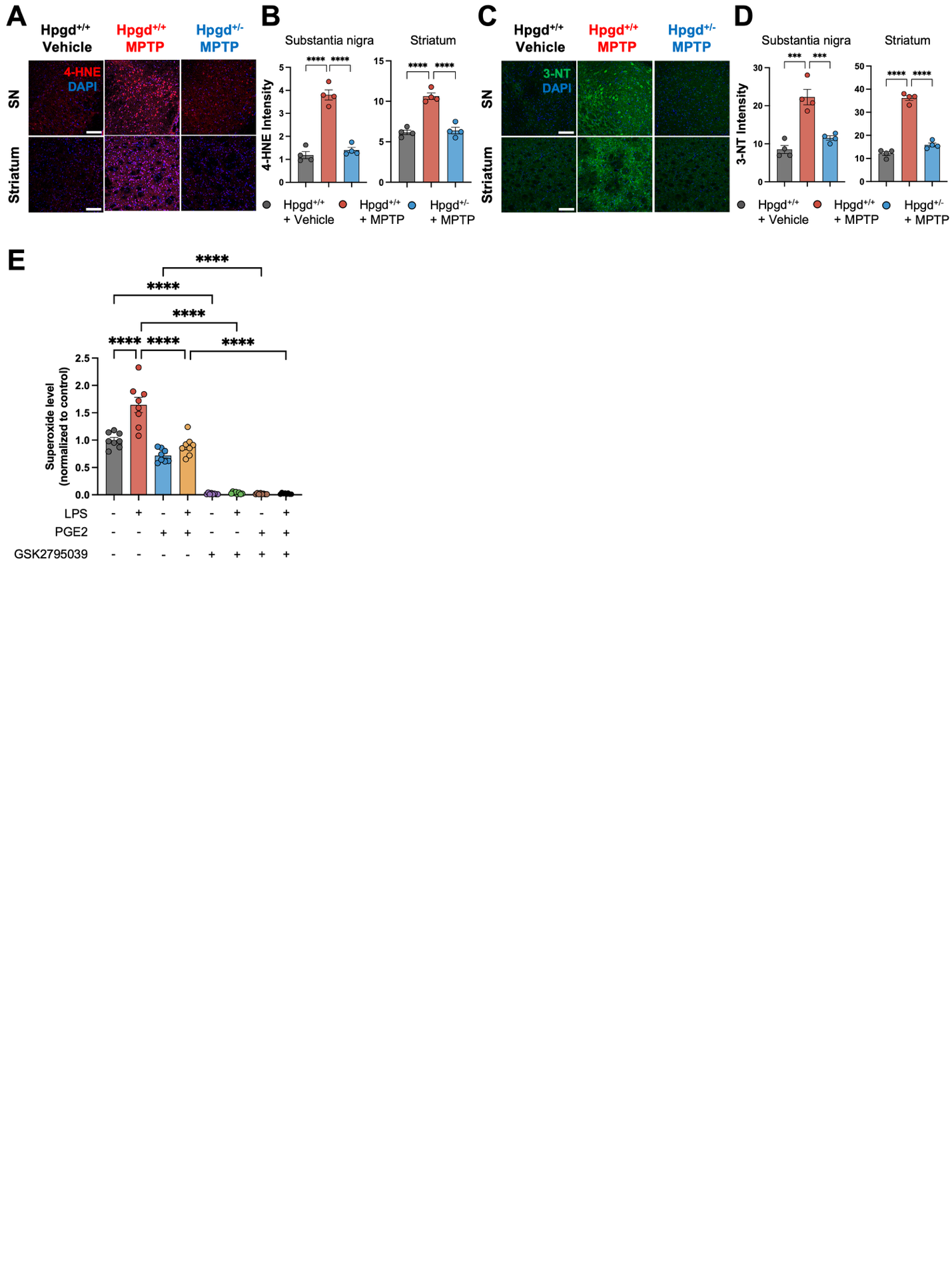
**

**Fig. S4. 15-PGDH substrates reduce NOX2 expression and ROS production in LPS-stimulated BV2 cells**

(A) Representative 4-HNE stained images of the substantia nigra and striatum of *Hpgd* heterozygous mice and their wild-type littermates treated with vehicle or MPTP (scale bar = 100 µm).

(B) Quantification of 4-HNE fluorescence signal shows that MPTP-treated *Hpgd* heterozygous mice have lower mean fluorescence intensity than their wild-type littermates in the substantia nigra and striatum (n=4 per group, **** p < 0.0001, one-way ANOVA and Tukey’s post hoc analysis).

(C) Representative 3-NT stained images of the substantia nigra and striatum of *Hpgd* heterozygous mice and their wild-type littermates treated with vehicle or MPTP (scale bar = 100 µm).

(D) Quantification of 3-NT fluorescence signal shows that MPTP-treated *Hpgd* heterozygous mice have lower mean fluorescence intensity than their wild-type littermates in the substantia nigra and striatum (n=4 per group, *** p < 0.001, **** p < 0.0001, one-way ANOVA and Tukey’s post hoc analysis).

(E) LPS-induced superoxide production in BV2 microglial cells was completely abolished by the NOX2 inhibitor GSK2795039 (n=8 per group, **** p < 0.0001, one-way ANOVA with Tukey’s post hoc analysis).

**
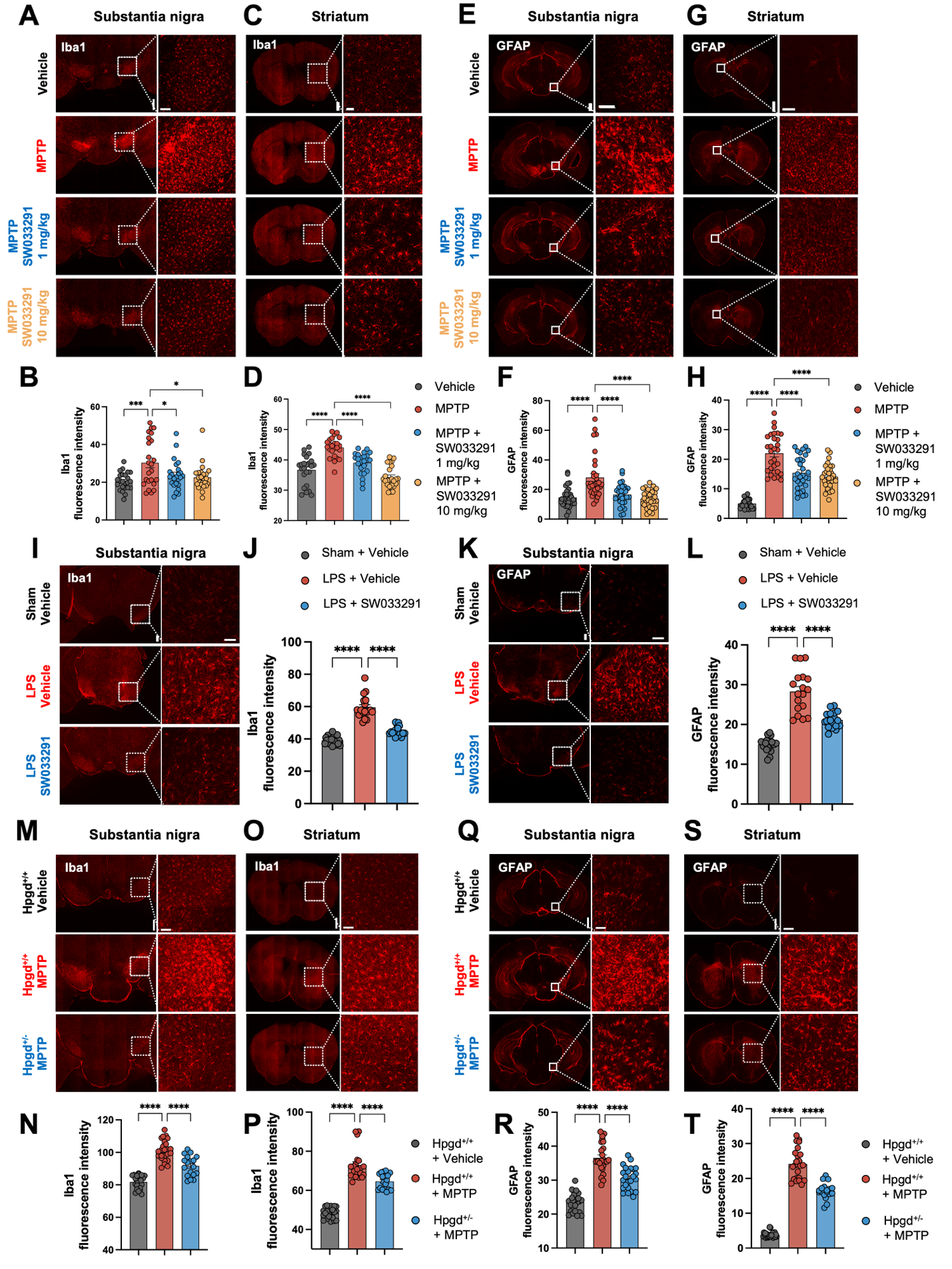
**

**Fig. S5. Pharmacological and genetic inhibition of 15-PGDH suppresses micro/astrogliosis in animal models of PD**

(A) Representative Iba1 stained images of the substantia nigra of mice treated with vehicle or MPTP in the absence or presence of SW033291 (scale bar = 600 µm for original image, 100 µm for inset).

(B) Quantification of Iba1 fluorescence signal shows that SW033291 treatment significantly reduced activated microglia in the substantia nigra of mice exposed to MPTP (n=4 per group, * p < 0.05, *** p < 0.001, one-way ANOVA and Tukey’s post hoc analysis)

(C) Representative Iba1 stained images of the striatum of mice treated with vehicle or MPTP in the absence or presence of SW033291 (scale bar = 800 µm for original image, 50 µm for inset).

(D) Quantification of Iba1 fluorescence signal shows that SW033291 treatment reduced activated microglia in the striatum of mice exposed to MPTP (n=4 per group, **** p < 0.0001, one-way ANOVA and Tukey’s post hoc analysis).

(E) Representative GFAP stained images of the substantia nigra of mice treated with vehicle or MPTP in the absence or presence of SW033291 (scale bar = 800 µm for original image, 100 µm for inset).

(F) Quantification of GFAP fluorescence signal shows that SW033291 treatment significantly reduced reactive astrocytes in the substantia nigra of mice exposed to MPTP (n=4 per group, **** p < 0.0001, one-way ANOVA and Tukey’s post hoc analysis).

(G) Representative GFAP stained images of the striatum of mice treated with vehicle or MPTP in the absence or presence of SW033291 (scale bar = 800 µm for original image, 100 µm for inset).

(H) Quantification of GFAP fluorescence signal shows that SW033291 treatment reduced reactive astrocytes in the striatum of mice exposed to MPTP (n=4 per group, **** p < 0.0001, one-way ANOVA and Tukey’s post hoc analysis).

(I) Representative Iba1 stained images of the substantia nigra from mice treated with vehicle or LPS, in the absence or presence of SW033291 (scale bar = 200 µm for original images, 50 µm for inset)

(J) Quantification of Iba1 fluorescence signal shows that SW033291 reduced Iba1 intensity in the substantia nigra of mice subjected to intranigral LPS administration (n=3 per group, **** p < 0.0001, one-way ANOVA and Tukey’s post hoc analysis).

(K) Representative GFAP stained images of the substantia nigra from mice treated with vehicle or LPS, in the absence or presence of SW033291 (scale bar = 200 µm for original images, 50 µm for inset)

(L) Quantification of GFAP fluorescence signal indicates that SW033291 reduced GFAP intensity in the substantia nigra of mice subjected to intranigral LPS administration (n=3 per group, **** p < 0.0001, one-way ANOVA and Tukey’s post hoc analysis).

(M) Representative Iba1 stained images of the substantia nigra of *Hpgd* heterozygous mice and their wild-type littermate treated with vehicle or MPTP (scale bar = 600 µm for original image, 100 µm for inset).

(N) Quantification of Iba1 fluorescence signal shows that MPTP-injected *Hpgd* heterozygous mice have reduced activated microglia in the substantia nigra compared to wild-type littermate (n=3-4 per group, **** p < 0.0001, one-way ANOVA and Tukey’s post hoc analysis).

(O) Representative Iba1 stained images of the striatum of *Hpgd* heterozygous mice and their wild-type littermate treated with vehicle or MPTP (scale bar = 600 µm for original image, 100 µm for inset).

(P) Quantification of Iba1 fluorescence signal shows that MPTP-injected *Hpgd* heterozygous mice have reduced activated microglia in the striatum compared to wild-type littermate (n=3-4 per group, **** p < 0.0001, one-way ANOVA and Tukey’s post hoc analysis).

(Q) Representative GFAP stained images of the substantia nigra of *Hpgd* heterozygous mice and their wild-type littermate treated with vehicle or MPTP (scale bar = 800 µm for original image, 100 µm for inset).

(R) Quantification of GFAP fluorescence signal shows that MPTP-injected *Hpgd* heterozygous mice have reduced reactive astrocytes in the substantia nigra compared to wild-type littermate (n=3-4 per group, **** p < 0.0001, one-way ANOVA and Tukey’s post hoc analysis).

(S) Representative GFAP stained images of the striatum of *Hpgd* heterozygous mice and their wild-type littermate treated with vehicle or MPTP (scale bar = 800 µm for original image, 100 µm for inset).

(T) Quantification of GFAP fluorescence signal shows that MPTP-injected *Hpgd* heterozygous mice have reduced reactive astrocytes in the striatum compared to wild-type littermate (n=3-4 per group, **** p < 0.0001, one-way ANOVA and Tukey’s post hoc analysis)

**
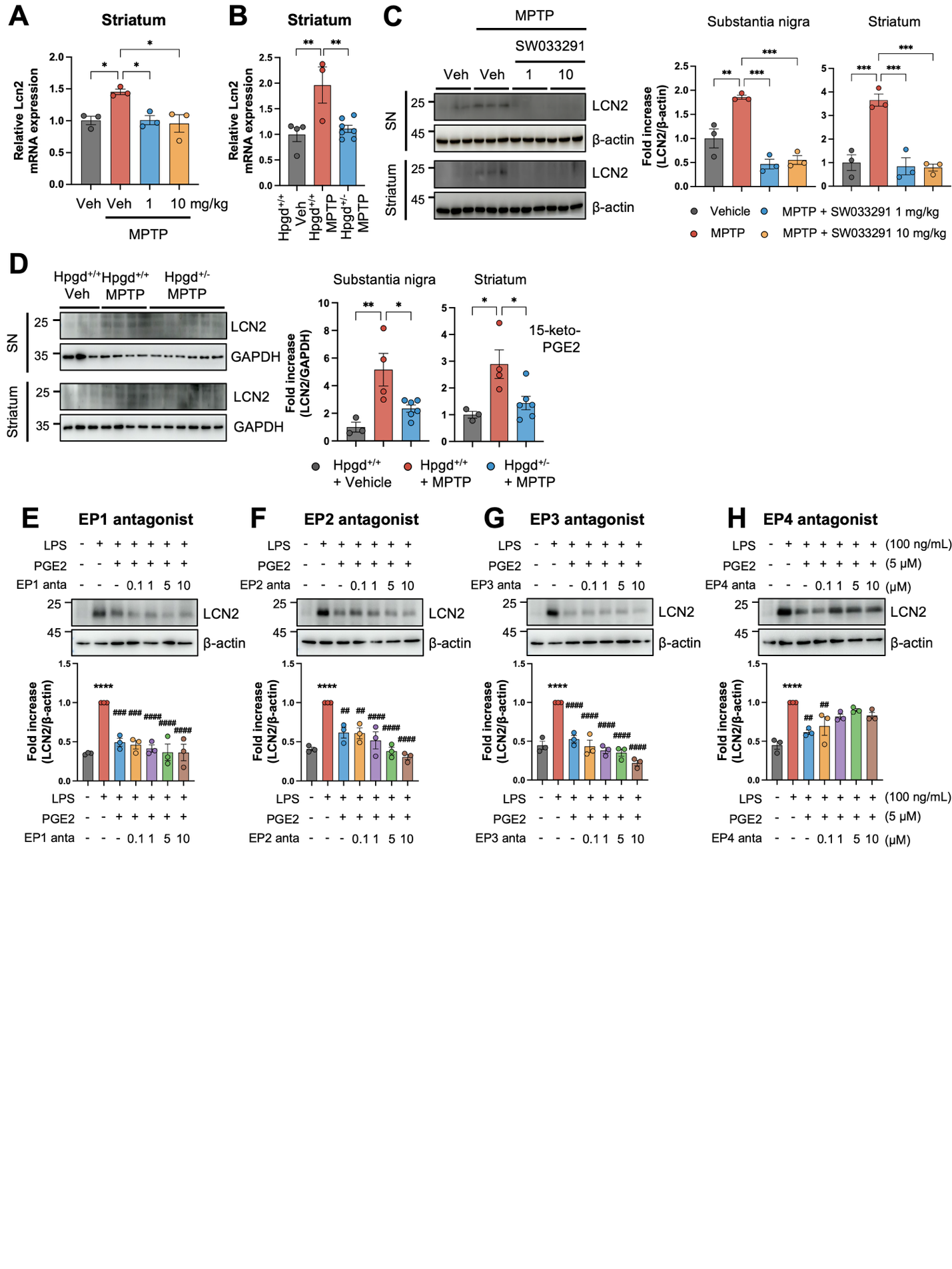
**

**Fig. S6. Inhibiting 15-PGDH reduces LCN2 expression through PGE2-EP4 axis**

(A) RT-qPCR analysis shows that *Lcn2* is upregulated in MPTP-treated striatum and that this increase is attenuated by treatment with 1 and 10 mg/kg of SW033291 (bars denoted 1 and 10 mg/kg) (n=3 per group, * p < 0.05, one-way ANOVA and Tukey’s post hoc analysis).

(B) RT-qPCR analysis shows that *Hpgd* heterozygous mice have lower *Lcn2* levels than their wild-type littermate treated with MPTP in the striatum (n=3-6 per group, ** p < 0.01, one-way ANOVA and Tukey’s post hoc analysis).

(C) Western blot and its quantification show that SW033291 significantly reduced LCN2 expression in the substantia nigra and striatum of MPTP-treated mice (n=3 per group, ** p < 0.01, *** p < 0.001, one-way ANOVA and Tukey’s post hoc analysis).

(D) Western blot and its quantification show that *Hpgd* heterozygous mice exhibit lower LCN2 expression in the substantia nigra and striatum of MPTP-treated mice compared to their wild-type littermates (n=3-6 per group, * p < 0.05, ** p < 0.01, one-way ANOVA and Tukey’s post hoc analysis).

(E) Western blot and its quantification show that pretreatment with EP1 antagonist did not block the effect of PGE2 on LPS-induced LCN2 up-regulation in BV2 cells (n=3 per group, *** p < 0.001, **** p < 0.0001, one-way ANOVA and Tukey’s post hoc analysis). For figures F-I, graphed values correspond to the western blot conditions directly above.

(F) Western blot and its quantification show that pretreatment with EP2 antagonist did not block the effect of PGE2 on LPS-induced LCN2 up-regulation in BV2 cells (n=3 per group, ** p < 0.01, *** p < 0.001, **** p < 0.0001, one-way ANOVA and Tukey’s post hoc analysis).

(G) Western blot and its quantification show that pretreatment with EP3 antagonist did not block the effect of PGE2 on LPS-induced LCN2 up-regulation in BV2 cells (n=3 per group, **** p < 0.0001, one-way ANOVA and Tukey’s post hoc analysis).

(H) Western blot and its quantification show that pretreatment with EP4 antagonist blocked the effect of PGE2 on LPS-induced LCN2 up-regulation in BV2 cells (n=3 per group, ** p < 0.01, **** p < 0.0001, one-way ANOVA and Tukey’s post hoc analysis).


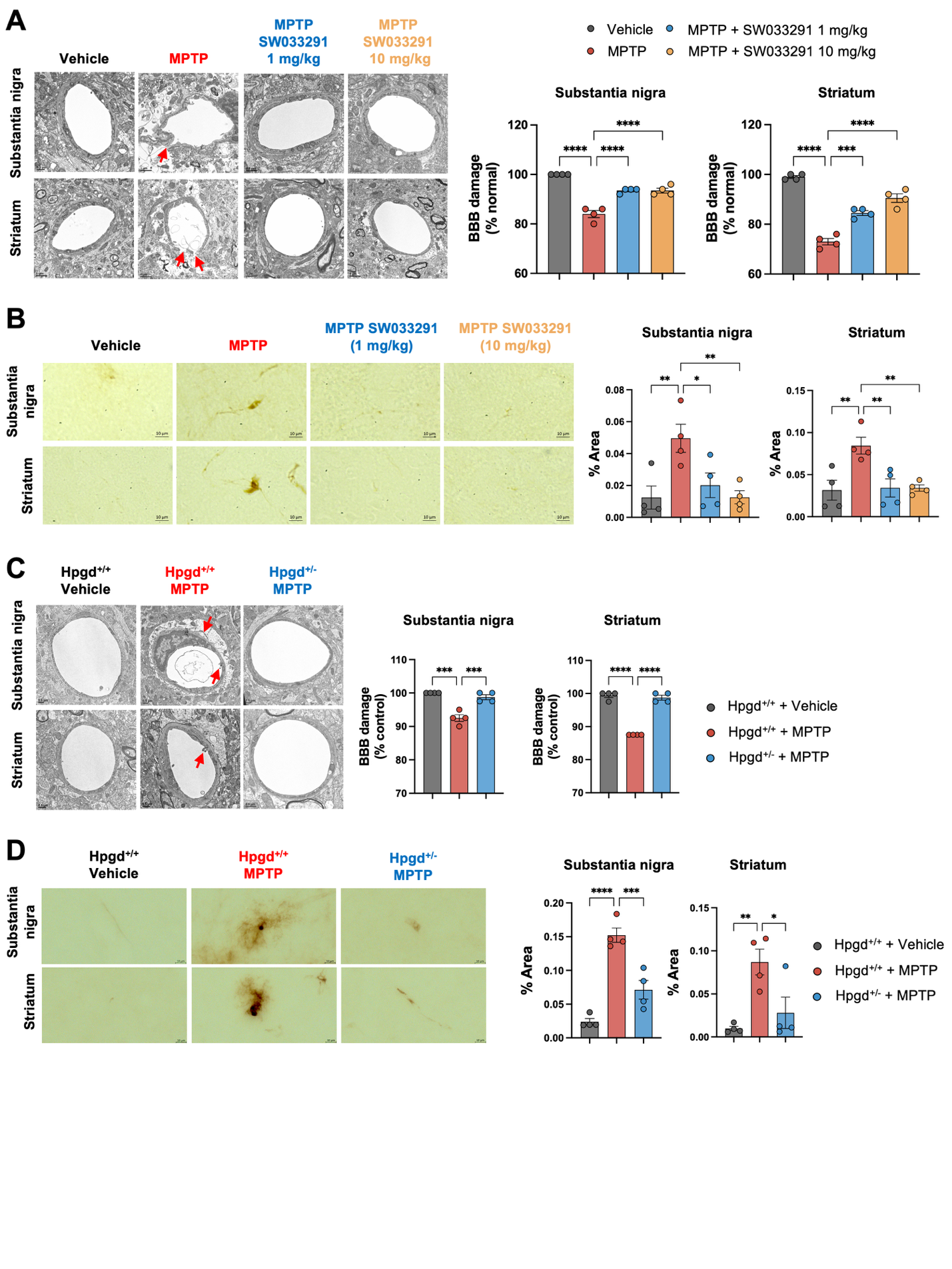


**Fig. S7. Inhibiting 15-PGDH protects from BBB disruption in the MPTP PD model**

(A) Representative transmission electron microscopy images of the BBB and their quantification demonstrate that SW033291 significantly protected against capillary endothelium breaks (red arrow) in the substantia nigra and striatum of MPTP-treated mice compared to vehicle-treated controls (scale bar = 0.5 and 1 µm, n=4 per group, *** p < 0.001, **** p < 0.0001, one-way ANOVA and Tukey’s post hoc analysis).

(B) Representative IgG-stained images and their quantification show that SW033291 treatment significantly reduced IgG infiltration in the substantia nigra and striatum of MPTP-treated mice compared to vehicle-treated controls (scale bar = 10 µm, n=4 per group, * p < 0.05, ** p < 0.01, one-way ANOVA and Tukey’s post hoc analysis).

(C) Representative transmission electron microscopy images of the BBB and their quantification demonstrate that *hpgd* haploinsufficient mice are protected against MPTP-induced capillary and astrocyte end-feet disruptions in substantia nigra and striatum (scale bar = 0.5 µm, n=4 per group, *** p < 0.001, **** p < 0.0001, one-way ANOVA and Tukey’s post hoc analysis).

(D) Representative IgG-stained images and their quantification indicate that *hpgd* haploinsufficient mice exhibit significantly reduced IgG infiltration in the substantia nigra and striatum of MPTP-treated mice compared to their wild-type littermates (scale bar = 10 µm, n=4 per group, * p < 0.05. ** p < 0.01, *** p < 0.001, **** p < 0.0001, one-way ANOVA and Tukey’s post hoc analysis).


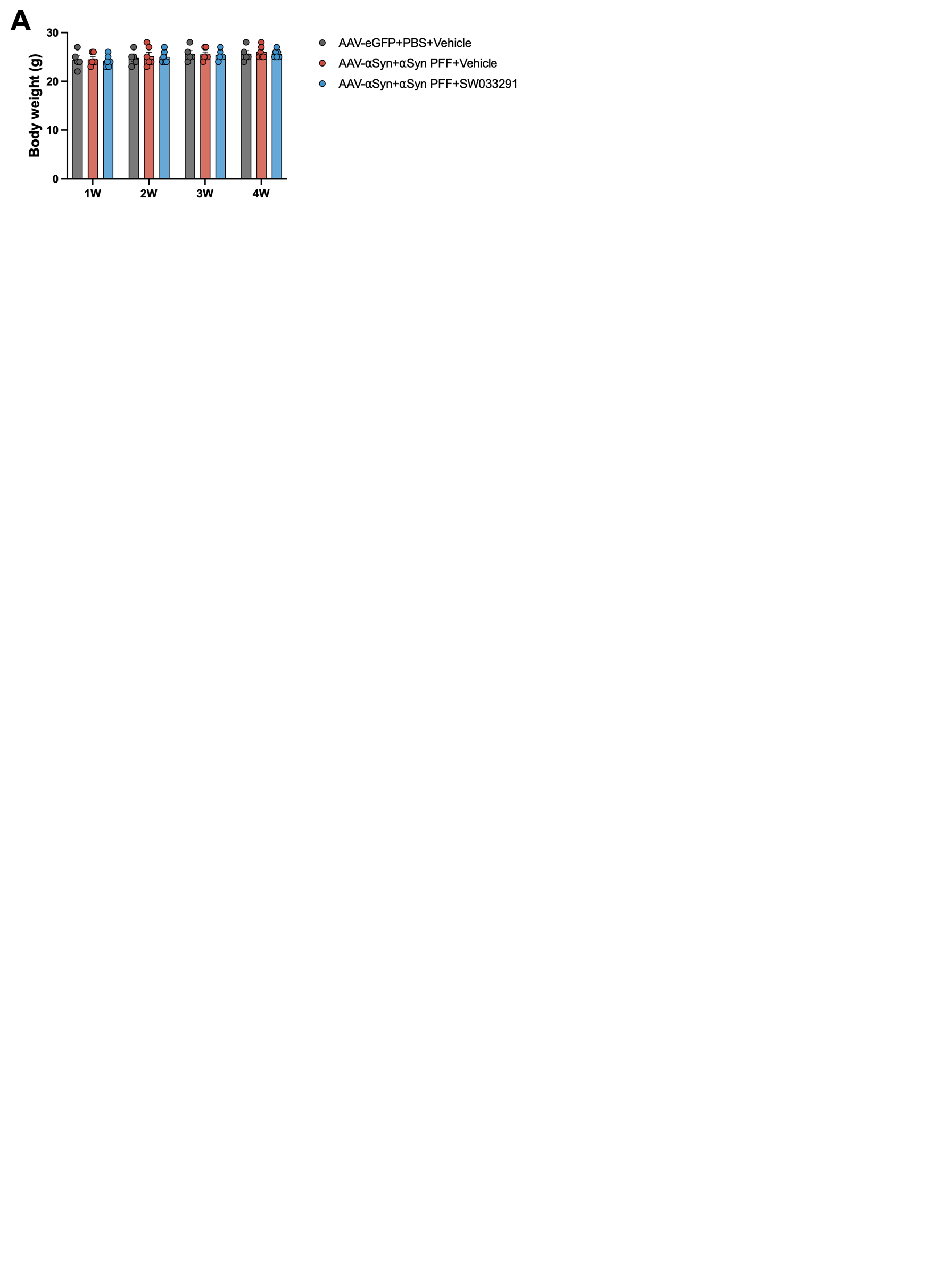


**Fig. S8. Inhibition of 15-PGDH has no effect on body weight in an α-synuclein mouse model**

(A) Body weight was not affected by SW033291 treatment in an α-synuclein mouse model (n=5-6 per group).

**Supplementary Table 1.** Demographic information of study subjects with PD.

**Supplementary Table 2.** Differentially expressed genes identified by bulk RNA sequencing of substantia nigra samples from MPTP or intranigral LPS mice treated with vehicle or SW033291.

Column A: ENSEMBL ID

Columns B-M: Normalized expression values for each sample

Column N: Gene symbol

Columns O-T: DESeq2 output - baseMean, log2FoldChange, lfcSE, stat, p-value (raw), padj (adjusted by Benjamini-Hochberg method)

Columns U-AF: Raw read counts for each sample

Columns AG-AR: VST-normalized expression values for downstream analysis

**Supplementary Table 3.** GO analysis associated with SW033291 upregulated genes (p<0.05 and Log2(FC)>0). Pathways are sorted in order of p value. Highlighted pathways are graphically depicted in Figure 4B.

Columns A-B: GO term ID and description

Columns C-F: GeneRatio, Bgratio, RichFactor, FoldEnrichment – Ratios and enrichment metrics

Column G: Standardized enrichment score

Columns H-J: Statistical significance values (p-value, BH-adjusted p-value, and q-value)

Columns K-L: Contributing gene IDs and their counts

**Supplementary Table 4.** GO analysis associated with SW033291 downregulated genes (p<0.05 and Log2(FC)<0). Pathways are sorted in order of p value. Highlighted pathways are graphically depicted in Figure 4C. The top 40 most significant pathways are annotated to identify those containing *Cybb*, *Cyba*, *Il1β*, and *Lcn2*.

Columns A-B: GO term ID and description

Columns C-F: GeneRatio, Bgratio, RichFactor, FoldEnrichment – Ratios and enrichment metrics

Column G: Standardized enrichment score

Columns H-J: Statistical significance values (p-value, BH-adjusted p-value, and q-value)

Columns K-N: Presence of genes in the gene IDs column for the top 40 most significant pathways (*Cybb, Cyba, Il1β*, and *Lcn2*)

Columns O-P: Contributing gene IDs and their counts
